# Parenting Practices and Externalizing Behaviors in Autistic Children: A Systematic Literature Review

**DOI:** 10.1007/s10567-024-00467-6

**Published:** 2024-02-26

**Authors:** Vedanta Suvarna, Lara Farrell, Dawn Adams, Lisa-Marie Emerson, Jessica Paynter

**Affiliations:** 1 School of Applied Psychology, Griffith University, Gold Coast, QLD Australia; 2 Autism Centre of Excellence, School of Education and Professional Studies, Griffith University, Mt Gravatt, QLD Australia; 3 School of Health Sciences, University of Canterbury, Christchurch, New Zealand

**Keywords:** Autism, Parenting, Children, Systematic review, Externalizing behaviors

## Abstract

There is limited recent research on the association between parenting practices and externalizing behaviors in autistic children. To address this gap, the current systematic review examined the associations between parenting practices and externalizing behaviors in autistic children, along with the mediating and moderating effects of parent and child variables (PROSPERO registration number CRD42022268667). Study inclusion criteria were (1) Peer-reviewed journals, (2) Participants included parents of autistic children and their children, (3) Quantitative measures of both parenting practices or behaviors/style and child externalizing behaviors, (4) Cross-sectional or longitudinal studies only, and (5) Studies published in English. Study exclusion criteria were: (1) Qualitative studies, (2) Published in a language other than English, (3) Participants included non-human participants, (4) Participants that did not include parents and their autistic children as participants or did not report this group separately, (5) Systematic review and meta-analyses, and (6) No quantitative measures of parenting practices and/or child externalizing behaviors. Quality appraisal and risk of bias were conducted using the McMaster Tool and results were synthesized in Covidence and Excel. Thirty studies were included in the review. Results demonstrated that mindful parenting was associated with fewer or lower levels of externalizing behaviors; positive parenting practices had non-significant associations with externalizing behaviors; specific parenting practices had differing associations with externalizing behaviors; and negative parenting practices were associated with higher levels of externalizing behaviors. We are unable to draw causal relationships due to focus on cross-sectional and longitudinal articles only. The potential for future research to target specific parent practices to support children’s externalizing behaviors is discussed.

**Supplementary Information:**

The online version contains supplementary material available at 10.1007/s10567-024-00467-6.

## Introduction

Autistic children perceive and engage with the world in a different way to their non-autistic peers, including having different ways of communicating with others, having highly focused interests, and engaging in self-stimulating behavior (Williams & Roberts, 2018). These children may also experience mental health challenges, including difficulties with anxiety, mood, and somatization (Crane et al., 2019; O'Nions et al., 2018; Ozsivadjian et al., 2021), sensory overload, pressure to mask their differences (Higgins et al., 2021; Strömberg et al., 2022), and challenges with transitions or unexpected changes (Sterling-Turner & Jordan, 2007). Taken together, these factors may relate to elevated rates of internalizing and externalizing  behaviors (O'Nions et al., 2018). Internalizing behaviors include symptoms associated with anxiety, mood conditions, and somatization (Lindsey et al., 2020; Smith et al., 2021). We use the term ‘externalizing behaviors’ to refer to ‘behavior(s) of such an intensity, frequency or duration that the physical safety of the person or others may likely be placed in serious risk, or behavior which may limit the use of, or result in the individual being denied access to ordinary public facilities’ (Emerson, 2001, p. 44). Externalizing behaviors include behaviors that others may find challenging (e.g., aggression, non-compliance, tantrums) (Bader et al., 2015; Schiltz et al., 2018) and are conceptualized as observable behaviors that relate to the person’s external environment (McRae et al., 2019). Parents of autistic children may make extensive adaptations to daily routines in response to their child’s externalizing behaviors (O'Nions et al., 2018, 2020). These accommodations may help support the child to engage in essential daily activities (e.g., reducing uncertainty, material rewarding). However, some accommodations may inadvertently maintain or exacerbate the challenges that the child faces and perpetuate externalizing behaviors (Lucyshyn et al., 2004). The purpose of this review is to investigate what parenting practices are associated with lower or higher levels of externalizing behaviors in autistic children. Understanding these behaviors and associated factors, specifically parenting practices is vital given externalizing behaviors are associated with suicidal ideation, depressive and anxiety symptoms, challenging parent–child interactions, and negative impacts on academic performance, at least within neurotypical children (Kerr et al., 2013; Soto-Sanz et al., 2019) with emerging research suggesting similar findings for autistic children (La Buissonnière Ariza et al., 2021).

### Parenting Styles and Parenting Behaviors in Parents of Autistic Children

Parenting refers to the coordination of behaviors, resources, and efforts that parents use to guide the growth, welfare, health, and behaviors of their children (Aguirre-Dávila et al., 2023). Parenting includes *parenting styles* and *parenting behaviors*. *Parenting styles* describe ‘how parents parent’ and have been defined along two broad dimensions: parental warmth and behavioral/psychological control (Lambrechts et al., 2011; Maljaars et al., 2014). *Parental warmth* includes *parenting behaviors* such as acceptance, involvement, sensitivity, and positive affect (also called *positive parenting*, see Maljaars et al., 2014; McRae et al., 2019). *Parental behavioral control* includes behaviors such as managing/controlling child behaviors via monitoring, setting limits and standards, strict discipline, adapting the environment, and *parental psychological control* includes more intrusive controlling behaviors such as inconsistent discipline, withdrawal of love and guilt induction (also called *negative parenting* in the literature) (Lambrechts et al., 2011; Maljaars et al., 2014). As a function of the two parenting dimensions of parental warmth and control, Baumrind’s *Theory of Parenting Styles* identified three parenting styles in parents of neurotypical children: permissive (high warmth and low control), authoritarian (low warmth and high control), and authoritative (high warmth and high control) (Baumrind, 1966, 2016). Thus, *parenting styles* are defined as a broad collection of behaviors expressed by the parent, which create an emotional environment for the child (Locke & Prinz, 2002). In recent research, mindful parenting has been introduced as an additional parenting style informed by mindfulness-based theory. It is based on Indian-Buddhist philosophy and defined as ‘paying attention to your child and your parenting in a particular way: intentionally, here and now, and non-judgmentally’ (Kabat-Zinn & Kabat-Zinn, 1997, p. 22). O’Nions et al. (2018) described other specific and observable parenting strategies (e.g., accommodating the child, modifying the environment), which are goal and response oriented to child behaviors (objective to reduce child behaviors or are responses to child behaviors). Taken together, parenting is a complex construct. Parenting research on neurotypical children suggests that it may be problematic to only include one type of behavior, style, or strategy as this may not encapsulate the full intricacy of parenting (Calders et al., 2019) or be representative of the larger population and in this case—the autistic community. Therefore, for the purpose of this review, the term *parenting practices* will be used, which is inclusive of the broader parenting styles, mindful parenting, and parenting behaviors that occur across parenting dimensions, such as warmth and control, and specific parenting strategies to support child behaviors.

### Associations Between Parenting Practices and Externalizing Behaviors

Specific parenting practices (e.g., parental control) have been associated with higher child externalizing behaviors in autistic children. McRae et al. (2018) found that a parenting practice characterized by harsh and disengaged parenting, inconsistent discipline, the use of corporal punishment, and poor monitoring and supervision, predicted higher levels of child externalizing behaviors. Further, Madarevic et al. (2022) found that parenting practices characterized as criticizing and ignoring the child were associated with greater externalizing behaviors in autistic children. Of note, however, across this research is that only one study (O’Nions et al., 2020) used an autism-specific measure of parenting to explore the relationship. This impacts interpretation as measures that are not designed for autistic children may not adequately capture parenting experiences for this population or may result in elevated scores where autism characteristics are rated as externalizing behaviors, thus, inflating associations (see discussion in Adams et al., 2019).

Other parenting practices, including mindful parenting have been associated with reduced child externalizing behavior in autistic children. Singh et al. (2006) found that mothers' increased mindful parenting was associated with their child’s decreased externalizing behaviors (such as aggression and non-compliance) and increased maternal self-efficacy in a small-scale multiple baseline study with three parent–child dyads. Similarly, Ridderinkhof et al. (2018), reported reductions in child externalizing behaviors, changes in parenting skills, and overall increased parental mindfulness following a mindful parenting intervention in a sample of 23 parent–child dyads. Taken together, these results suggest increased mindful parenting, may be associated with lower levels of child externalizing behavior, at least within parent intervention research.

O’Nions et al.’s (2018) meta-synthesis explored qualitative data on parenting practices in response to externalizing behaviors in autistic children. The authors found a range of parenting strategies used by parents of autistic children to support their child’s behaviors, including accommodating the child, modifying the environment, providing structure and routine, supervision and monitoring, managing non-compliance with everyday activities, responding to externalizing behaviors, managing distress, maintaining safety, and analyzing and planning. This study found that accommodating the child’s needs appeared to be the most common strategy used by parents in response to externalizing behaviors. This review was limited to qualitative analysis of case studies, case series, and qualitative studies, and as such could not investigate the strength of associations between parenting practices and externalizing behaviors. To improve outcomes for autistic children and their parents, it would be beneficial to understand and synthesize the quantitative research on associations between parenting practices and externalizing behaviors of autistic children to inform future parent-focused interventions.

### Aim and Research Questions

The primary aim of this review is to synthesize the current literature on the associations between parenting practices among parents of autistic children and child externalizing behaviors through a systematic quantitative literature review. Evidence for the mediating and/or moderating role of other parent variables (e.g., parenting stress) and child variables (child age and gender) on the association between parenting practices and child externalizing behaviors will also be explored. Given limited understanding within the autism field on child age and gender effects on parenting practices (Maljaars et al., 2014), the literature will also be reviewed to address this gap to gain an understanding of possible child age and child gender effects on parenting practices. Understanding potential mediators or moderators of associations between parenting practices and child behaviors may be of value to identify families at higher probability of challenges, or targets for supports (e.g., reduction of parent stress may facilitate more supportive parenting).

The research questions are:

1. What is the strength of association between parenting practices and child externalizing behaviors in autistic children?
2. What is the scope and quality of research examining the associations between parenting practices and child externalizing behaviors among samples of autistic children?
3. Do any parent or child variables have mediating or moderating effects on the association between parenting practices and child externalizing behaviors?
4. Does child age and/or sex/gender have mediating or moderating effects on the associations between parenting practices and child externalizing behaviors?

## Method

The protocol for this review is registered on the PROSPERO database (registration number CRD42022268667). Variations to the protocol included moving from the terminology of ‘challenging behavior’ to *externalizing behavior* and ‘on the autism spectrum’ to *autistic* in line with preferred language by the autistic community (Monk, 2022). This systematic review was conducted in accordance with the Preferred Reporting Items for Systematic Review and Meta-Analyses checklist (PRISMA; Page et al., 2021).

### Search Strategy and Study Selection

PROSPERO was first searched to check if other systematic reviews on this topic existed. The search strategy was then developed by the first author in consultation with the university librarian and reviewed by the co-authors. The search strategy had three main categories: the target population—autistic child; second target population and their parenting practices—parents of autistic children and their parenting styles and behaviors; and child behaviors—externalizing behaviors exhibited by the autistic child, see Supplementary Material Table 2 for full listing.

### Eligibility Criteria

Studies were identified via electronic searches of PsycINFO, CINAHL, PUBMED, Scopus, and Web of Science. Inclusion criteria were: (1) Published in peer-reviewed journal, (2) Participants included parents of autistic children (no restrictions on autistic children’s age, i.e., adult children will be included), or subgroups of this population, (3) Included a quantitative measures of parenting practices and externalizing behaviors of the autistic child, (4) Cross-sectional or longitudinal design, and (5) published in English. No publication date restrictions. Exclusion criteria were: (1) Qualitative studies, (2) Studies published in a language other than English, (3) Studies that included non-human participants, (4) Studies that did not include parents and their autistic children as participants or did not report this group separately, (5) Systematic review and meta-analyses, and (6) No measure of parenting practices and/or child externalizing behaviors.

### Quality Assessment

Quality appraisal of selected studies was conducted using the McMaster Quality appraisal tool—McMaster Critical Review Form for Quantitative Studies (Law et al., 1998), which included: study purpose clarity, literature review relevance, appropriateness of study design, sampling, outcome measure reliability and validity, results analysis and interpretation. Inter-rater was conducted by a second reviewer (see Acknowledgements) rating 20% of the articles (see Supplementary Material Table 4) which resulted in 94% agreement.

### Data Extraction and Management

Data were extracted and managed by the first author within Covidence (Covidence, 2022). Information extracted included: parenting practices and child externalizing behaviors and strength of associations between constructs. Where reported, data (measure, impact) on moderators and mediators of this association by other parent variables (e.g., parenting stress effects) and child variables (age and gender/sex of child) were also extracted. Other data extracted included: authors, year/location, participants numbers, sex/gender, ethnicity, age of child and parents, child autism diagnosis, child co-occurring conditions, parent mental health status and/or diagnoses, socio-economic status, parent type, measures used, and effect sizes found.

### Data Synthesis

Data synthesis involved creating categories addressing the research questions and collating the key information from eligible studies, see Table 1. Parenting practices were grouped into those that were associated with lower child externalizing behaviors, mixed outcomes, and higher levels of child externalizing behaviors.

**Table 1:** Characteristics of included studies

References	Country	Race/ethnicity	Child age (years; *M*_*age*_ or Range)	Child* n* and gender	Parent age (years; *M*_*age*_ or Range)	Parent *n* and gender	Measures	
							Parenting practices	Child externalizing behaviors
Aydin (2022)	Turkey	NA	3–6	*n* = 273; 205 males (75%), 51 females (19%), 17 did not wish to specify (6%)	31–40	221 mothers, 45 fathers, 7 did not wish to specify	MIPQ (Total score)	PKBS-2 (Total score)
Bader and Barry (2014)	USA	88% White, 5% Black, 4% Latino, 3% Mixed or Other ethnicity	8–18	Time 1 gender not specified Time 2 *n* = 84; 73 (87%) Males, (11) 13% females	32–58	Time 1 *n* = 111; 96% mothers, 4% fathers	APQ scale (Positive composite—Parental involvement and Positive parenting; Negative composite—Poor monitoring/supervision, Inconsistent discipline, and Corporal punishment)	CBCL (Composite score—Rule-breaking and Aggressive subscales)
Bader et al. (2015)	USA	91% White, 4% Black, 3% Latino, and 2% Mixed or Other ethnicity	6–18	*n* = 111; 95 (86%) Male, 16 (14%) female	25–58	*n* = 111, 97% Mothers	APQ (Positive composite—Parental involvement and Positive parenting; Negative composite—Poor monitoring/supervision, Inconsistent discipline, and Corporal punishment)	CBCL (Composite score—Rule-breaking and Aggressive subscales)
Beer et al. (2013)	Australia	85.7% White	3–20	*n* = 28 24 (85.7%) Males, 4 females	32–76	*n* = 28; 4 Males, 24 females (all biological parents, except 1 = grandparent)	IM-P (Total score)	Problem Behaviors subscale of NCBR (Total score)
Berliner et al. (2020)	USA	79.1% White, 13.4% Latino, 5.9% Black, 5.9% Native American, 1.5% Asian/Pacific Islander	2.5–4.5	*n* = 67; 51 Males (76%) and 16 females (24%)	20–54	*n* = 67; 59 Females and rest males	PS subscales (Laxness and Overreactivity)	ECBI (Total score)
Boonen et al. (2014)	Canada	NA	6–12	*n* = 206; 175 (85%) Males, 31 (15%) females	Mothers 31–52 Fathers 31–73	358 Mothers (93%), 35 fathers (7%)	PBS-A Composite scores (Positive parenting—Positive parenting, Material rewarding and Rules); Negative control (Discipline and Harsh punishment); Autism adapted parenting (Stimulating Development and Adapting the Environment)	SDQ (Composite score—Rule-Conduct and Hyperactivity subscales)
Brinkman et al. (2022)	USA	90% White	5–15	*n* = 120; 95 Males (79%), 25 females (21%)	26–54	*n* = 120; 90 Females, 30 males (116 biological parents)	FQ—Parental criticism and Emotional overinvolvement subscale scores	BASC-3 composite score—Externalizing: Hyperactivity, aggression, and conduct problems
Cheung et al. (2019)	Hong Kong	100% Chinese	*M*_*age*_ = 9.39	111 Boys (81.62%) and 25 girls (18.38%)	*M*_*age*_ = 43.09	*n* = 136; 111 Mothers (81.62%)	BMPS (Total score)	SDQ (Composite score—Problem behavior subscales: emotional symptoms, conduct problems, hyperactivity, and peer problems)
Clauser et al. (2021)	USA	NA	3–18	*n* = 70; 56 Males (80%) and 14 females (20%)	18–55	*n* = 70; 66 Mothers, 4 fathers	PSDQ subscale scores	CBCL (Composite score—Rule-breaking and Aggressive subscales)
Davies et al., 2022	UK	85.5% White	3–22 *M*_*age*_ = 11.50	*n* = 25 (Autism group); 20 (80%) male, 5 (20%) female	22–72, *M*_*age*_ = 40.81	*n* = 155; 150 females, 5 males	PCRI parenting behavior subscale scores only (Involvement, Communication, Limit Setting, Autonomy)	SDQ (Total score)
De Clercq et al. (2021)	Belgium	90% Mothers and 88.6% fathers Belgian, 9.3% mothers, 7.1% fathers Other European, 0.7% fathers Non-Europeans	Time 1 5.1–16.2 *M*_*age*_ = 10.1 Time 2 11.6–22.6 *M*_*age*_ = 16 Time 3 14.4–23.9 *M*_*age*_ = 19	Time 1 *n* = 140 Time 2 *n* = 97 Time 3 *n* = 116; Predominantly male all time points (83%) Time 3 96 males and 20 females	Time 1 *M*_*age*_ = 39.9	*n* = 141, Mainly mothers 98.6%	PBS subscale (Negative Control subscale scores—Discipline (described as punitive parenting) and Harsh punishment)	CBCL (Composite score—Rule-breaking and Aggressive subscales)
De Clercq et al. (2019)	Belgium	90.7% Belgian, 6.6% European non-Belgian, 2.7% non-European	7–15 *M*_*age*_ = 12.5	*n* = 95; 73 (77%) Males, 22 females (23%)	Mother *M*_*age*_ = 42.5, Father *M*_*age*_ = 45	*n* = 95; 94% were mothers	PCS; Autonomy Support Scale of the POPS	CBCL (Composite score—Rule-breaking and Aggressive subscales)
Dieleman et al. (2017)	Belgium	90.6% Mothers and 88.4% fathers Belgian, 8.7% mothers and 7.2% fathers Other European, 0.7% fathers Non-Europeans	Time 1 5.1–16.2 *M*_*age*_ = 10.2 Time 2 11.6–22.6 *M*_*age*_ = 16 Time 3 14.4–23.9 *M*_*age*_ = 19	5:1 Ratio, mostly males (24 females) Time 1 114 males (82%) and 24 females (18%)	Time 1 Mothers *M*_*age*_ = 39.9, Father *M*_*age*_ = 42.6	Time 1 *n* = 138; 98% mothers Time 2 *n* = 97 Time 3 *n* = 114	PBS composite scores (Positive parenting—Positive parenting subscale + Rules subscale; Negative control—Discipline and Harsh punishment)	CBCL (Composite score—Rule-breaking and Aggressive subscales)
Dieleman et al. (2018)	Belgium	85% Mothers and fathers Belgian, 8% mothers and 6% fathers Other European, 2% mothers and 4% fathers Other	14–24 *M*_*age*_ = 18.8	*n* = 95; 72 Males (76%), 23 females(24%)	Mothers *M*_*age*_ = 48.4, Father *M*_*age*_ = 51	*n* = 95; 96% are mothers	Psychological control subscale of PCS, Autonomy Support Scale of the POPS, Overreactivity scale of the Parenting Scale	CBCL (Composite score—Rule-breaking and Aggressive subscales)
Dieleman et al. (2019)	Belgium	100% Belgian	7–15 *M*_*age*_ = 10.92	29 Males (70.7%), 12 females (29.3%)	32–55 *M*_*age*_ = 41.84	41 Mothers only	Psychological control subscale of PCS, Autonomy Support Scale of the POPS	CBCL (Composite score—Rule-breaking and Aggressive subscales)
Greenlee et al. (2022)	USA	90.4% White, 9.6% Other	5–12 *M*_*age*_ = 7.92	*n* = 188; 160 Males (85.6%), 28 females (14.4%)	Mothers 24–54 *M*_*age*_ = 38.73, Father 22–60 *M*_*age*_ = 40.76	188 Couples (biological parent 88.4%)	PSDQ subscale scores	CBCL (Composite score—Rule-breaking and Aggressive subscales)
Lindsey et al. (2020)	USA	83.7% White, 4.7% Latino, 3.9% Black, 3.9% Bi/multiracial	Time 1 4–10 *M*_*age*_ = 6.87	*n* = 129; 102 Males (79%), 27 females (21%)	Time 1, 24–52 *M*_*age*_ = 34.67	*n* = 129; 94 Females, 35 males	APQ (Positive composite—Parental involvement and Positive parenting; Negative composite—Poor monitoring/supervision, Inconsistent discipline)	BASC-3 (Externalizing problems composite score)
Maljaars et al. (2014)	Belgium	NA	6–18 *M*_*age*_ = 12.1	*n* = 552; 303 (55%) Males, 249 (45%) females	NA	*n* = 552	PBS subscales, PBS-A autism subscales only	SDQ (Composite score—Conduct problems and hyperactivity)
McRae et al. (2018)	USA	47.8% White, 47.8% Black, 1.5% Latino, 3% Biracial	6–12 *M*_*age*_ = 9.58	*n* = 67; 59 (88%) Males, 8 females (12%)	NA	*n* = 67; 64% Mothers, 7% fathers	APQ (Warmth/supportive composite—Parental involvement and Positive parenting; Harsh/disengaged composite—Poor monitoring/supervision, Inconsistent discipline, and Corporal punishment)	CBCL (Composite score—Rule-breaking and Aggressive subscales)
McRae et al. (2019)	USA	50% Black, 46% White, 2% Latino, 2% Biracial	6–12 *M*_*age*_ = 9.52	*n* = 50; 46 (92%) Males, 4 (8%) females	NA	*n* = 50; 64% Mothers, 7% fathers	APQ (Warmth/supportive composite—Parental involvement and Positive parenting; Harsh/disengaged composite—Poor monitoring/supervision, Inconsistent discipline, and Corporal punishment)	CBCL (Composite score—Rule-breaking and Aggressive subscales)
Mills et al. (2022)	USA	66% White, 5% Black, 5% Latino, 2% Southeast Asian, 2% West Asian, 8% Multiethnic, 2% preferred not to disclose	8–13 *M*_*age*_ = 9.70	*n* = 44; 37 Males (84%), 7 females (16%)	29–54	40 Mothers	BMPS (Total score)	ERC (Lability/Negativity Subscale score)
O’Nions et al. (2020)	UK	NA	6–16 *M*_*age*_ = 11.1	156 Males (70%), 64 females (29%), and 2 other gender id (1%)	40–44	*n* = 222, 214 Mothers, rest other	PSQ subscale scores PBS-A subscale scores (items adjusted post exploratory factor analysis) and APQ (total score)	EDI Reactivity, HSQ-Autism and EDA-Q subscale scores
Osborne et al. (2008)	UK	NA	5–16	*n* = 72; 70 Males (97%), 2 females (3%)	NA	72 Parents	PCRI parenting behavior subscale scores only (Involvement, Communication, Limit Setting, Autonomy)	SDQ (Composite score—Difficulties/Problem behavior subscales: emotional symptoms, conduct problems, hyperactivity, and peer problems)
Portes et al. (2020)	Brazil	NA	3–7 *M*_*age*_ = 59.07 months	*n* = 45; 39 Males (86.7%), 7 females (13.3%)	Mothers *M*_*age*_ = 31.78, Father *M*_*age*_ = 36.36	*n* = 90, 45 Mothers, 45 fathers	PSDQ (Portuguese father and mother versions) subscale scores	SDQ (Brazilian) Total score
Rahman and Jermadi (2021)	Malaysia	NA	4–12	*n* = 79; 49 Males (62%), 30 females (38%)	30–54	*n* = 79; 49 Mothers (62%) and 30 fathers (30%)	PSDQ subscale scores	SDQ subscales (reported separately)
Raulston et al. (2021)	USA	80% White, 5% Bi/multiethnic, 15% Other	*M*_*age*_ = 7.80	*n* = 75; 63 (84%) Males, 12 females (16%)	*M*_*age*_ = 39.30	*n* = 75; 91% Mothers	BMPS (Total score)	SDQ conduct subscale score
Shawler and Sullivan (2017)	USA	90.8% White	3–11 *M*_*age*_ = 8.57	*n* = 130; 116 Males (89.2%), 14 females (10.8%)	24–58 *M*_*age*_ = 39.81	*n* = 130; 115 Mothers (88.5%), 11 fathers (8.5%), 4 adopted mothers (3%)	PS Total score = Laxness + Overreactivity subscales; PS Laxness and Overreactivity subscales	ECBI Total score
Storch et al. (2015)	USA	NA	NA	*n* = 40; 33 Males (82.5%), 7 females (17.5%)	NA	*n* = 40 Parents	PAS subscale scores (Frequency, Parent Impact, and Child Impact subscales)	CBCL Externalizing subscale score
Ueda et al. (2020)	USA	100% Japanese	6–12 *M*_*age*_ = 4.10	*n* = 42; 29 Males (69%), 13 females (31%)	32–48 *M*_*age*_ = 40.64	42 Mothers	PSDQ subscale scores (Japanese translation, four corporal punishment items from Authoritarian subscale were deleted)	CBCL (Japanese) Externalizing subscale score
Ventola et al. (2017)	USA	NA	*M*_*age*_ = 9.35 years	*n* = 48; 24 Males (50%), 24 females (50%)	NA	NA	PRPBI Total scores and Acceptance, Psychological control subscale scores	CBCL (Composite score—Rule-breaking and Aggressive subscales)

*APQ* Alabama Parenting Questionnaire (Shelton et al., 1996), *BASC-3* The Behavior Assessment System for Children–Parent Rating Scale-Third Edition–Preschool and Child Versions (Reynolds & Kamphaus, 2004), *BMPS* Bangor Mindful Parenting Scale (Jones et al., 2014), *CBCL* Child Behavior Checklist (Achenbach, 1991), *ECBI* Eyberg Child Behavior Inventory (Eyberg & Ross, 1978), *EDA-Q* The Extreme Avoidance Questionnaire (O'Nions et al., 2014), *EDI* The Emotion Dysregulation Inventory (Mazefsky et al., 2018), *ERC* Emotion Regulation Checklist (Shields & Cicchetti, 1997), *FQ* Family Questionnaire (Wiedemann et al., 2002), *HSQ-Autism * Home Situations Questionnaire (Chowdhury et al., 2016), *IM-P* Interpersonal Mindfulness in Parenting Scale (Duncan, 2007), *MIPQ* Mindfulness in Parenting Questionnaire (Gördesli et al., 2018), *NCBR* Nisonger Child Behavior Rating Form (Aman et al., 1996), *PAS* Paediatric Accommodation Scale (Benito et al., 2015), *PBS* Parental Behavior Scale (Van Leeuwen & Vermulst, 2004), *PBS-A* The Parental Behavior Scale-ASD (Van Leeuwen & Noens, 2013), *PCRI* Parent–Child Relationship Inventory (Gerard, 1994), *PCS* Psychological Control Scale (Barber, 1996), *PKBS-2* Preschool and Kindergarten Behavior Scale (Merrell, 2002), *PS* Parenting Scale (Arnold et al., 1993), *POPS* Perceptions of Parents Scale (Grolnick et al., 1991), *PRPBI* Parent Report of Parenting Behavior Inventories (Galejs & Pease, 1986), *PSDQ* The Parenting Styles and Dimensions Questionnaire (Robinson et al., 2001), PSDQ The Parenting Styles and Dimensions Questionnaire Portuguese (Pedro et al., 2015), *PSQ* Parenting Strategies Questionnaire (O’Nions et al., 2018), *SDQ* The Strengths and Difficulties Questionnaire (Goodman, 1997)

## Results

**Fig. 1:**
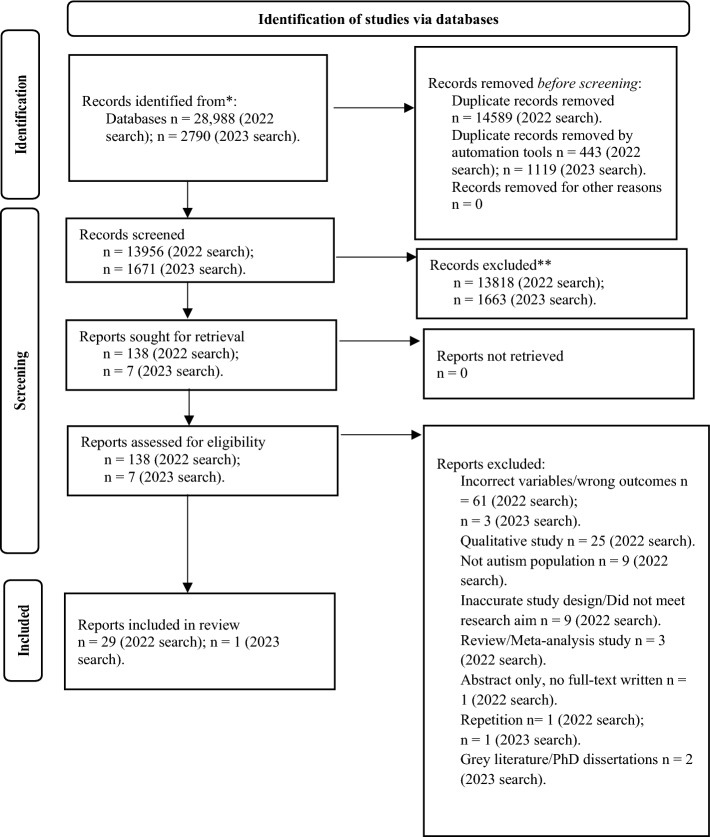
PRISMA flow chart of articles screened

Searches were conducted on 25/04/2022 and updated on 10/03/2023. A total of 28,988 (2022 search) + 2790 (2023 search) (see Fig. 1) studies were identified, 13,956 (2022 search) + 1671 (2023 search) after removal of duplicates, which were screened by title/abstract. This resulted in 138 (2022 search) + 7 (2023 search) full-text articles being assessed for eligibility through full-text review, of which 29 articles (2022 search) + 1 article (2023 search) met full criteria. Five studies were subsequently excluded as they did not explore the associations between parenting practices and child externalizing behaviors, part of the inclusion criteria. Therefore, in total, 30 studies met all inclusion criteria (see Fig. 1). See Table 1 for participant characteristics and study characteristics of included studies. A second reviewer (see acknowledgements) screened 10% of the titles and abstracts of the articles found (selected using a random number generator). There was 98.93% agreement on papers meeting the inclusion criteria and 100% agreement on papers meeting the exclusion criteria. Discrepancies were discussed with a third reviewer (author four) until consensus was reached. This suggested high reliability of the inclusion/exclusion criteria for selected studies. Overall agreement was substantial (Cohen’s *κ* = 0.72). Although, this review focused on externalizing behaviors in autistic children, data from studies that included a wide age range of children in their sample (e.g., 1–23.9 years) were retained (*n* = 3).

### Child Gender, Age, and Ethnicity Information

Child participants were predominantly male, with most studies (*n* = 29) reporting child gender in a binary format (one study did not report gender). Across these there were 2480 males, 770 females, and two other gender, and 17 no answer/declined, with an overall ratio of 3.4:1 male:female. The child age range varied from 2.5 to 24 years old. Only 19 out of the 30 included studies reported the race/ethnicity of participants (see Table 1), with the majority of participants categorized as White (approximately 86%). Of these 19 studies, 2 studies included 100% Asian participants, and another 2 studies had a similar ratio of Black and White participants.

### Risk of Bias and Quality of Studies

The risk of bias of the included studies was assessed (Supplementary Material Table 4). All studies included were considered low risk for selective reporting, implying that all included outcome measures were analyzed and reported. Twenty-four studies used a cross-sectional design, six studies had a longitudinal design. Most studies used reliable measures and only three studies (Boonen et al., 2014; Maljaars et al., 2014; O’Nions et al., 2020) used autism-specific measures. The included studies had varied definitions for parenting practices and externalizing behaviors (see definitions). All studies reported that they had approval from an appropriate ethical body for data collection and informed consent was indicated.

#### Variability Across the Research

##### Definitions

The definition of child externalizing behaviors varied among the included studies with some studies (*n* = 6) (e.g., Portes et al., 2020; Shawler & Sullivan, 2017) including a range of behaviors (e.g., externalizing behaviors included internalizing behaviors within the definition, such as anxiety related symptoms), whereas others defining externalizing behaviors or specific externalizing behaviors (e.g., aggression) (e.g., Boonen et al., 2014; Storch et al., 2015). In terms of parenting practices, there were a range of definitions [i.e., parenting dimensions and behaviors (positive—warmth dimension, or negative behaviors—control dimension); parenting styles (authoritative, authoritarian, permissive); mindful parenting; specific parenting strategies (accommodation, reducing uncertainty)]. The definitions on mindful parenting practices were consistent in the five studies (i.e., Aydin, 2022; Beer et al., 2013; Cheung et al., 2019; Mills et al., 2022; Raulston et al., 2021) investigating mindful parenting. Definitions of parenting styles (authoritative, authoritarian, permissive) (e.g., Clauser et al., 2021; Greenlee et al., 2022) were consistent across the studies. Definitions of specific parenting behaviors (positive, negative, warm, supportive, lax, autonomy, control) (e.g., Bader & Barry, 2014; Brinkman et al., 2022; Dieleman et al., 2018; McRae et al., 2018) were similar across studies. Definitions of parenting strategies to support externalizing behaviors (e.g., reinforcement, reducing uncertainty, accommodation) (i.e., Boonen et al., 2014; Maljaars et al., 2014; O’Nions et al., 2020; Storch et al., 2015) differed across the studies, i.e., different operational definitions of parenting strategies were used, with differences observed even for specific strategies, e.g., O’Nions et al. (2020) defined accommodation as particular observable behaviors specific to parents of autistic children, whereas Storch et al. (2015) defined accommodation as behaviors adopted by parents to reduce child behaviors. Three of the four studies used the PBS-A measure to measure these parenting strategies, with the exception of Storch et al. (2015) who used the PAS scale.

##### Measurement

All 30 studies used self-report measures to measure parenting practices (see Supplementary Material Table 3 for parenting classification scheme) and parent reports to measure child externalizing behaviors. Most of the measures used in the included studies were not specifically designed/validated for parents of autistic children (*n* = 27/30 studies). Exceptions were O’Nions et al. (2020) who used autism-specific measures for both parenting practices (PSQ and PBS-Autism) and child behaviors (HSQ-Autism), and Maljaars et al. (2014) and Boonen et al. (2014) who used the PBS-Autism to measure parenting practices. Six studies reported a total/subscale child behaviors score (which included items that assessed internalizing behaviors as well). Two studies reported a total score of a measure evaluating externalizing behaviors only. Twenty two of 30 studies reported on child externalizing behaviors separately (i.e., subscales/composite scores).

### Associations Between Parenting Practices and Externalizing Behaviors in Autistic Children

#### Parenting Practices Associated with Lower Levels of Externalizing Behaviors in Autistic Children

##### Mindful Parenting Practices

Five studies reported on mindful parenting practices measured using MIPQ, IM-P, and BMPS (Supplementary Material Table 7). All studies used a subscale of child externalizing behaviors, except Aydin et al. (2022) who used a total score of PKBS-2 scale. All five studies identified mindful parenting as negatively associated with child externalizing behaviors (*r* =  − .26 to − .42), i.e., higher levels of mindful parenting were associated with lower levels of child behavior (using composite measures of intensity and frequency). Four of the five studies recruited school-aged children, one study (Aydin et al., 2022) investigated this association in 3- to 6-year-old children. All five studies found similar findings with no age or gender differences across studies.

#### Parenting Practices and Mixed Associations with Externalizing Behaviors in Autistic Children

##### Parenting Practices: Parenting Styles (Authoritarian, Authoritative, Permissive)

Five studies reported on the association between parenting styles (e.g., as authoritative, authoritarian, and permissive styles) and child externalizing behaviors (frequency) using the PSDQ to measure parenting styles (see Supplementary Material Table 5). Results were consistent across studies for authoritarian parenting with higher levels of authoritarian parenting associated with higher levels of externalizing behaviors (frequency). Results for permissive parenting were relatively consistent with four studies finding higher levels of permissive parenting style associated with higher levels of externalizing behaviors (frequency). One exception, however, was Rahman and Jermadi (2021) who found no significant associations. Rahman and Jernadi (2021) study differed in that participants were Malaysian, and majority of the parents rated their parenting style as authoritative (highest *M* and use) and majority of the recruited participants had autistic children with a medical condition (87%), thus, adopting a parenting style with high warmth and high control, which may explain differing results.

Findings for authoritative parenting style were mixed. Greenlee et al. (2022) found in a large longitudinal study that higher levels of authoritative parenting at Time 2 in fathers were associated with lower levels of autistic child externalizing behaviors (frequency) at Time 3 one year later (*r* =  − .30**). However, in contrast, higher levels of authoritative parenting at Time 2 in mothers  were associated with higher levels of externalizing behaviors at Time 3 (*r* = .22*). This finding for mothers was similar to Portes et al. (2020), who found cross-sectionally that higher levels of authoritative parenting in mothers and fathers were  associated with higher levels of externalizing behaviors (frequency) in autistic children and Clauser et al. (2021) who found that authoritative parenting in mothers and fathers contributed to higher levels of externalizing behaviors (frequency). In contrast, two studies (Rahman & Jermadi, 2021; Ueda et al., 2020) found null effects. Of note, these latter two studies differed in that they used subscale scores instead of composite scores and included Malaysian and Japanese participants in contrast to participants from the USA or Brazil.

##### Parenting Practices: Parenting Strategies to Support Externalizing Behaviors

Four studies reported on parenting practices—parenting strategies to support their child’s externalizing behaviors (Supplementary Material Table 6). These included the broader concepts of—accommodating the child, modifying the environment, providing structure and routine, supervision and monitoring, managing non-compliance with everyday activities, responding to externalizing behaviors, managing distress, maintaining safety, and analyzing and planning, and autism-adapted behaviors. These are discussed together as they represent parenting strategies implemented to support their child’s externalizing behaviors. Three studies by Boonen et al. (2014), Maljaars et al. (2014), and Storch et al. (2015) found no associations between parenting practices (autism-adapted parenting, material rewarding, adapting the environment, stimulating the environment, and accommodation, respectively) and externalizing behaviors. O’Nions et al. (2020), found positive associations between parenting practices that included accommodation behaviors and four specific child externalizing behaviors (reactivity, demand-specific non-compliance, extreme demand avoidance, and social inflexibility non-compliance) with greater parental accommodation related to higher levels of child behaviors in both intensity and frequency (*r* = .41 to .53). Parenting practices such as reducing uncertainty had positive associations, i.e., were associated with higher levels, with three specific child externalizing behaviors (reactivity, extreme demand avoidance, and social inflexibility non-compliance) (*r* = .15 to .22), whereas reinforcement approaches had a positive association, i.e., associated with higher levels of, with only extreme demand avoidance, *r* = .13*. Overall, results suggested that higher levels of the specific parenting practices of accommodation are associated with higher levels of specific child externalizing behaviors (both intensity and frequency). Differences between the three studies might have been due to specific subscales used by O’Nions et al. (2020), which were context sensitive and autism adapted, with other studies using either the SDQ composite scores (Boonen et al., 2014; Maljaars et al., 2014) or CBCL composite scores (Storch et al., 2015), which were not specifically designed for autistic children. Mixed findings could also be due to different sample sizes, e.g., Boonen et al. (2014), Maljaars et al. (2014) and O’Nions et al. (2020) had relatively large sample sizes and recruited similar aged group children, whereas Storch et al. (2015) had a smaller sample size.

##### Parenting Practices: Warmth Dimension

Twelve studies (Supplementary Material Table 6) reported parenting practices along the warmth dimension, described as positive parenting, parenting involvement, communication, autonomy/ autonomy-supportive, satisfaction, and warm/supportive. Nine studies found no associations between warmth dimension parenting practices and externalizing behaviors. These studies had a mix of cross-sectional and longitudinal designs and used the following measures: APQ, PBS-A, PBS, PCRI, and Autonomy Support subscale. However, four studies found significant but contrasting associations. A cross-sectional study by Boonen et al. (2014) found that an element of warmth- positive parenting had a small association (*r* = .16) with more externalizing behaviors (frequency). A longitudinal study by Dieleman et al. (2017) found that an element of warmth- positive parenting, was associated one year later (*r* = .33) with more externalizing behaviors (frequency). In contrast, a longitudinal study Osborne et al., (2008) found another element of warmth involvement, was associated with fewer externalizing behaviors (frequency). Similarly, a cross-sectional study by Brinkman et al. (2022) found that overinvolvement was associated with higher levels of externalizing behaviors (frequency). Taken together, the relationship between parental warmth and child externalizing behaviors is unclear, potentially due to the way warmth is operationalized (e.g., positive parenting vs. involvement vs. overinvolvement) and the way it is measured impacting any association with externalizing behaviors.

##### Parenting Practices: Control Dimension

Eighteen studies (Supplementary Material Table 6) reported parenting practices along the control dimension, including negative parenting, negative control, psychological control, criticism, parenting overreactivity, limit setting, harsh/disengaged, discipline, rules, and harsh punishment. Across these studies, the association ranged between *r* =  − 0.6 and 0.63. Specifically, most (14 studies) found higher levels of controlling parenting practices (i.e., negative parenting, psychological control, parenting overreactivity, harsh/disengaged parenting, discipline, rules, and harsh punishment) were associated with higher levels of child externalizing behaviors (frequency). Only two studies (Davies et al., 2022; Osborne et al., 2008) found that higher levels of a specific control parenting practice–limit setting were associated with lower levels of child externalizing behaviors (frequency). These studies may have found different associations due to being the only studies that analyzed the specific parenting practice of limit setting (measured with PCRI Limit setting scale). It should be noted that both studies found a strong significant association; however, both studies were limited due to their measure of child behavior including internalizing behaviors, which likely heightened the strength of the associations between parenting practices and child behaviors. The 14 studies that reported a positive association  between parenting practices along the control dimension and child externalizing behavior used composite scores/subscales (*n* = 12/14) or total scores (*n* = 2/14) of externalizing behaviors. Two studies used total scores of a specific measure of externalizing behaviors only, i.e., Berliner et al. (2020) and Shawler and Sullivan (2017) used total scores of the ECBI measure and its associations with PS total and subscale scores.

Four studies reported on parenting practices—discipline with conflicting findings. Maljaars et al. (2014) found significant positive associations with externalizing behaviors (frequency) and higher levels of discipline (*r* = .25*). In contrast, O’Nions et al. (2020) found significant positive associations with two specific externalizing behaviors and inconsistent discipline using a different measure (demand-specific *r* = .22*** and extreme demand avoidance* r* = .24***) (frequency and intensity). Similarly, Berliner et al. (2020) found that lax discipline also had a significant positive effect on child externalizing behaviors (frequency), suggesting that more lax parenting behaviors were associated with higher levels of externalizing behaviors. However, a large study (*n* = 130) by Shawler and Sullivan (2017) on parenting laxness/lax discipline in parents of children aged between 3 and 11 years, reported a non-significant finding. It should be considered this latter study used a non-autism-specific measure to measure parenting practices. Further, Berliner et al. (2020) recruited participants with children between the ages of 2.5 and 4.5 years and may have also found an association due to a smaller sample size (i.e., potentially inflated associations).

### Mediating and Moderating Effects

#### Parent Variables: Parenting Stress, Parenting Practices, and Parent Well-Being

Two studies (Cheung et al., 2019; Osborne et al., 2008) explored mediating and moderating effects of parenting stress on the association between parenting practices and child externalizing behaviors. Using longitudinal data, Osborne et al., (2008) found that parenting stress mediated the relationship between parenting practices at baseline and child externalizing behaviors at follow-up, such that higher levels of parenting practices (i.e., limit setting) were associated with lower levels of parenting stress, and in turn were associated with lower levels of child externalizing behaviors. Cheung et al. (2019), analyzed the mediating and moderating effects of parenting stress on the association between parent variables (mindful parenting, parents’ affiliate stigma, and parents’ mental well-being) and child externalizing behaviors. Parenting stress mediated the relationship between mindful parenting and child externalizing behaviors, such that higher levels of mindful parenting were associated with lower levels of parenting stress, and lower levels of parenting stress were associated with lower levels of child externalizing behaviors. The moderating effects of parenting stress on the above relationship was non-significant.

Parent variable moderators/mediators of relationship between parenting practices and child externalizing behaviors were rarely explored in the included studies. Instead, parenting practices were explored as the mediator/moderator on parent variables in six studies (see Supplementary Material Table 8). These studies found mediating and/or moderating effects of parenting practices, such that specific parenting practices had significant effects on child externalizing behaviors. Aydin (2022) found partial mediating effects of mindful parenting on the relationship between parental emotional regulation difficulties and child externalizing behaviors. Greenlee et al. (2022) found that authoritarian parenting styles mediated the relationship between parenting relationship satisfaction and child externalizing behaviors. Lindsey et al. (2020) found moderating effects of negative parenting practices, such that when negative parenting practices were higher, the association between autism characteristics and externalizing behaviors had a significant negative relationship; however, lower levels of negative parenting practices had no moderating effects, i.e., non-significant association between autism characteristics and externalizing behaviors. Osborne et al., (2008) found that the association between parenting stress at baseline and child externalizing behaviors at follow-up was no longer present, when the parenting practice of limit setting mediated the relationship. Shawler and Sullivan (2017) found that the parenting practice of discipline mediated the relationship between parenting stress and frequency of child externalizing behaviors, specifically parenting practice of harsh and overreactive behaviors mediated the relationship between parenting stress and frequency of child externalizing behaviors. One other study (Cheung et al., 2019) analyzed the moderating effects of parent well-being and found no significant effects.

##### Child Variables: Age and Gender

Child’s age was controlled for in eight studies, and child gender was controlled for in seven studies (i.e., these were entered stepwise and not analyzed as mediators or moderators). Among studies, there were generally no age or gender effects found, with one exception of Maljaars et al. (2014). They explored the effects of child age on parenting practices and found that specific parenting practices, including adapting the environment, were reported at a lower levels for adolescents as compared to children. Further, they found that material rewarding was lower for adolescents as compared to younger children. Only one of 30 studies analyzed the moderating effects of child gender on the association between parenting practices and child externalizing behaviors (Dieleman et al., 2017). The authors found that gender did not moderate the relationship between parenting practices (negative control and positive parenting) and child externalizing behaviors.

## Discussion

The aim of this review was to synthesize the quantitative evidence on parenting practices and their relation to externalizing behaviors in autistic children. Key findings were: (1) Mindful parenting was associated with lower levels of child externalizing behaviors, such that as mindful parenting increased, child externalizing behaviors decreased; (2) There were mixed results for other parenting practices—parenting styles, parenting strategies, and practices across warmth and control dimensions; (3) There is significant variability in operationalizing and measuring key constructs, including child externalizing behaviors (e.g., total scores vs. subscales) and parenting practices (e.g., specific measure). The implications of these findings, limitations of the review, implications and conclusions are overviewed below.

All five studies on mindful parenting found that higher levels of mindful parenting were associated with lower levels of child externalizing behaviors. While these studies had a cross-sectional design and cannot determine causality, the broader intervention research on mindful parenting (Bögels et al., 2010; Burgdorf et al., 2019; De Bruin et al., 2015; Ridderinkhof et al., 2018), provides promising findings of a potentially causal relationship whereby increasing mindful parenting (via mindful parenting interventions) is associated with reduced child externalizing behaviors (Bögels et al., 2010; Burgdorf et al., 2019; De Bruin et al., 2015; Ridderinkhof et al., 2018). Furthermore, this research suggests that mindful parenting may be a potential target for interventions, considering that it is amenable to change and increases support for children as seen in previous research (e.g., Bögels et al., 2014; Ridderinkhof et al., 2018). Thus, there is potential for supporting autistic children via increasing their parent’s mindful parenting.

Autism literature on parenting practices—styles, parenting strategies, and behaviors across the warmth and control dimension showed mixed results on the associations with child externalizing behaviors. Although, all five studies that analyzed parenting styles used the same measure to analyze parenting styles, there were mixed findings between specific parenting styles [authoritative (high warmth and high control) and permissive (high warmth and low control)] and externalizing behaviors in autistic children. One common finding, however, was that authoritarian parenting styles (low warmth and high control) were associated with higher levels of child externalizing behaviors. This is consistent with research in neurotypical children and their parents (Calders et al., 2019). A meta-analysis by Pinquart (2017) with neurotypical children found bidirectional associations between externalizing behaviors and parenting behaviors of harsh and psychological control, as well as with behavioral control/authoritative parenting styles. Therefore, we cannot assume the direction of relationships, given this finding may be bidirectional or is based on associations and whether use of this parenting style exacerbates, challenges, or whether it is a helpful response in reaction to challenges autistic children face (e.g., difficulties with change or uncertainty that may lead to higher rates of externalizing behavior). The meta-analysis by Pinquart (2017) also found that higher levels of authoritarian parenting and parenting behaviors including harsh and psychological control, were associated with higher levels of externalizing behaviors concurrently and longitudinally, whereas parenting behaviors including warmth and behavioral control were associated with lower levels of externalizing behaviors concurrently and longitudinally. Autistic children may benefit from this parenting style or behaviors including behavioral control (e.g., require more proactive control from their parent in certain situations that they may find overwhelming/stressful), which may provide their children with more structure and/or to manage child externalizing behaviors, as seen in the neurotypical literature (Pinquart, 2017). This is consistent with the research on parenting training interventions for the autistic community (e.g., via parent-mediated interventions such as RUBI parent training), which provides promising findings of a potentially causal relationship whereby increasing proactive control parenting is associated with reduced child externalizing behaviors [see Randomized Controlled Trials (RCT) by Aman et al., 2009; Bearss et al., 2015]. This highlights the importance of parent-mediated interventions within the autism field to better support parents and their autistic children. Further, two studies that found no effects, used subscale scores instead of composite scores and included Malaysian and Japanese participants. Thus, measurement and/or cultural differences in parenting may impact associations observed between authoritative parenting and child externalizing behavior.

Parenting practices across the warmth dimension such as, warm/supportive parenting did not have any associations with child externalizing behaviors, with only two studies, one cross-sectional study (Boonen et al., 2014) finding a significant small positive association with child externalizing behaviors and a longitudinal study (Dieleman et al., 2017), finding a significant positive association between earlier child externalizing behaviors and positive parenting practices 1 year later. These results are in contrast to the broader parenting literature on neurotypical children that suggests protective effects of the warmth dimension on children’s behavior (e.g., Calders et al., 2019; Pinquart, 2017). It may be that autistic children respond differently to these practices or there are differences in parent–child synchrony impacting the benefits of parental warmth in certain situations (e.g., child requiring higher control/lower warmth in certain situations). Further changes over time indicate potential temporal effects, i.e., parents may adopt more warm parenting practices to address their child’s needs associated with their externalizing behaviors.

Findings on parenting practices—involvement/overinvolvement were mixed. Brinkman et al. (2022) found a significant positive association between the parenting practice of overinvolvement and child externalizing behaviors, whereas Osborne et al., (2008) found a significant negative association between the parenting practice of involvement and child externalizing behaviors. Osborne’s findings are consistent with the neurotypical literature, for example, Badri et al. (2014) found that the parenting practice of involvement (at school to help with transition from pre-school to kindergarten) was negatively associated with externalizing behaviors in neurotypical children. It is posited that differing findings between studies may indicate that the levels of parenting involvement and context might be important to consider, e.g., parent involvement during important transitions. However, higher levels of involvement may be unsupportive and become counter-productive and unsupportive for autistic children in some settings or at higher level as is seen in neurotypical development and may exacerbate externalizing behaviors.

Further, there was evidence of moderate to strong positive associations between parenting practices across the control dimension (i.e., negative control, psychological control, overreactivity) and child externalizing behaviors, suggesting that as these parenting practices increase, child externalizing behaviors also increase (e.g., Dieleman et al., 2018; Ventola et al., 2017). However, some mixed findings were obtained with two studies (Davies et al., 2022; Osborne et al., 2008) finding higher levels of the parenting practice—limit setting was associated with lower levels of child externalizing behaviors. Both these studies used a measure that included internalizing behaviors; therefore, it is likely that these associations may not be the same for externalizing behaviors only. However, these results on parenting practices—limit setting are similar to other studies with other neurodivergent samples (e.g., Austin et al., 2004), suggesting that in contrast to control broadly, that limit setting may be helpful and associated with lower levels of externalizing behaviors in neurodivergent children (e.g., provision of expectations and structure via limit setting/discipline). The seemingly differing associations between individual parenting practices and child externalizing behavior indicated between studies may reflect the importance of context. Context-dependent interventions for externalizing behavior have long been associated with positive outcomes with autistic children, including behavior analytic-informed intervention approaches (e.g., Triple-P Program, Tellegen & Sanders, 2014; Whittingham et al., 2009) and parent-mediated behavioral interventions (see review by Postorino et al., 2017). The theoretical foundation of such approaches with autistic children is in understanding the function of any given behavior, and modifying contingencies to address that function and shape behavior. In contrast, mindfulness-based approaches, such as those included within this review, do not seek to determine the function of specific child behavior, or to target this within the intervention. Rather, the theoretical underpinnings of mindfulness-based approaches are grounded in Buddhist cultural understandings of human behavior and focus on intrapersonal processes (and inter-personal processes in the case of mindful parenting) rather than contextual factors. Within the parenting context, these interventions are parent-focused, and target intrapsychic changes of the parent (e.g., parenting stress) to help parents be more effective in supporting child behavior. The pathways of change in child behavior resulting from these interventions may be quite different to behavioral analytic approaches. Future research that aims to determine both efficacy as well as mediators of change associated with different parenting interventions will elucidate the nuances associated with the complexity of parenting and its influences on child behavior outcomes.

Studies that reported on discipline/parenting laxness found mixed findings. These studies recruited different age groups of autistic children and used different measures to measure key constructs. Berliner et al. (2020) found inconsistent discipline/lax discipline was associated with an increase in externalizing behaviors. However, somewhat contradicting these findings, Maljaars et al. (2014), who used an autism-specific measure to assess parenting practices, found that higher levels of rules and discipline were associated with higher levels of externalizing behaviors. Contrasting further, Shawler and Sullivan (2017) found no associations between these parenting practices—laxness and child externalizing behaviors. These mixed findings appear to be consistent with the literature on discipline/parenting laxness in parents of neurotypical children, where Marchand-Reilly (2012) found that lax discipline (measured with PS scale) in fathers was a significant predictor of child externalizing behaviors (child age 4–8 years old), while Park et al. (2018) found that lax discipline (measured with APQ) was uniquely associated with child behaviors for mothers but not fathers (child age 9–12 years old). Future autism-based studies could reconcile these results by investigating the differences in caregiver type and related associations, the impacts of child age on these associations, as well as use autism-specific measures to ensure to accurately capture behaviors in autistic children.

Further, four studies explored parenting strategies to support their child externalizing behaviors including accommodation, reducing uncertainty, rewarding, and autism-adapted behaviors with mixed findings. Three studies (Boonen et al., 2014; Maljaars et al., 2014; Storch et al., 2015) found no associations with externalizing behaviors, whereas O’Nions et al. (2020) found specific parenting strategies of reinforcement approaches were associated with lower levels of child externalizing behaviors, whereas accommodation and reducing uncertainty were associated with increased levels of child externalizing behaviors. This is consistent with research on parenting accommodation and an increase in child obsessive compulsive related difficulties and internalizing behaviors (Lebowitz et al., 2012). The differences between the included studies (i.e., associations found when using a measure validated for the autistic community and to measure specific strategies to manage behaviors in autistic children) highlights the importance of further research using appropriate autism outcome measures for all constructs of interest to ensure they are sensitive to this community. Further, this also highlights the importance of further research on potential target (i.e., parenting practices) for interventions.

Two studies found mediating effects of parenting stress, such that parenting practices such as limit setting was associated with higher parenting stress, and in turn was related to higher child externalizing behaviors (Osborne et al., 2008). Whereas, mindful parenting was associated with lower levels of parenting stress and consequently lower levels of child externalizing behaviors (Cheung et al., 2019). These findings are consistent with research in neurotypical development (Jackson & Choi, 2018) that have similarly found parenting stress is associated with parenting practices that lead to greater child challenges. Considering that studies have consistently indicated that parents of autistic children experience considerably higher levels of stress as compared to other groups (Barroso et al., 2018), and that parenting stress is amenable to intervention in this group (e.g., Bögels et al., 2010), further research in this area could identify potential factors to target in interventions, as well as help better understand this relationship, and therefore, an important avenue for further research. Six studies found mediating/moderating effects of parenting practices on the relationship between various parent and child variables (e.g., parenting stress, parent emotional regulation, parent relationship satisfaction, child autism characteristics) and child externalizing behaviors, highlighting a potential target (i.e., parenting practices) for interventions to mitigate the impact of for example parenting stress on child behavior.

Mediating or moderating effects of child age were not explored among the included studies. Only one study explored the moderating effects of gender and found no effects. Age and gender were controlled for in some studies. Considering that research suggests that autistic females may use more compensatory behaviors (‘camouflaging’) (Bitsika & Sharpley, 2019), gender differences in behaviors may moderate the association between parenting practices and child externalizing behaviors and, therefore, requires further research. Research also suggests that some behaviors may be shown more often by younger autistic children (e.g., eating difficulties, sleeping, dependency, emotional regulation), and may be associated with higher levels of stress in for parents (Davis & Carter, 2008), which may impact associations with outcomes. This highlights the consideration of the effects of these variables in future autism research.

Across studies, there was variability in the way parenting practices and child behaviors were operationalized and measured. Only three studies used autism-specific measures to evaluate parenting practices, of which only one study used autism-specific measures to evaluate both parenting practices and child externalizing behaviors. The other studies used a range of different measures of parenting practices and child externalizing behaviors, which are validated for neurotypical families and not neurodivergent families and, therefore, not likely representative of the autism community (e.g., specific parenting practices may be beneficial for autistic children to provide structure and consistency). Further, six of 30 included studies used total scores of child behaviors (therefore, including a broader range of behaviors). More inclusive research with increased specificity (e.g., using subscale scores) and autism-specific measures/methodology will lead to improved generalizability and practical application of findings (e.g., identify specific intervention targets that aligns with the goals of the autistic community). The varying definitions and measures of child and parent variables lead to a lack of specificity on definitions of key variables and highlight the need for clear definitions for parent and child variables within the autism field. Further, it is also important to factor that not all discipline practices are a ‘negative’ parenting practice (Berliner et al., 2020).

Studies included in this review were based on self-report data on perceived parenting practices and parent-report data on child externalizing behaviors by parents, and thus, subject to bias (e.g., social desirability bias on rating ones’ own parenting approach; as well as stressed and/or depressed parents likely to over-report levels of child externalizing behaviors). Future researchers could consider the use of multi-informant data to reduce biases associated with self-reports (e.g., teacher) and/or use clinician observed data as well. Children’s perceptions of their behaviors could also be considered to gain a broader understanding of parenting practices relationships with externalizing behaviors.

The review also identified the variability in the definitions and measures used to investigate key constructs within the autism field (e.g., parenting, child behaviors, externalizing, and internalizing behaviors). Only three studies used autism-specific measures. Future research could use autism-specific measures (e.g., The Autism Symptoms Dimension Questionnaire, Developmental Behavior Checklist, Parenting Strategies Questionnaire) to ensure the measures are sensitive to differences in child externalizing behaviors (e.g., is social avoidance due to the profile of autism or other difficulties). Future research could also use autism-specific measures that captures differences in severity of the externalizing behaviors to reduce associated ceiling effects that measures designed for neurotypical populations may not capture, as well as identify other externalizing behaviors not commonly seen in neurotypical children (e.g., echolalia) (Adams et al., 2019). This also highlights the importance of further research on appropriate and universal terminology for key constructs for this community. This will help increase the robustness of the literature and consequently supports for this community.

### Limitations

While this review addresses an important gap in the literature of synthesizing the quantitative research to date on the association between parenting practices and child externalizing behaviors a number of limitations are acknowledged. First, a choice was made a priori to focus on only quantitative articles. Qualitative studies and synthesis such as meta-synthesis may be valuable in future to provide in-depth understanding of particular behaviors (Robertson & Simmons, 2015), and important stakeholder perspectives (e.g., on the ‘why’). Second, only English language articles were included in this review, likely excluding several studies from non-English speaking countries/published in a different language, which may explain or at least contribute to the lack of diversity in participant ethnicity/race in reviewed studies. Future research could evaluate relationships between parenting practices and externalizing behaviors in autistic youth with more diverse populations, noting the homogeneity of the samples included in this review (86% of sample categorized as white). Third, to ensure manageable numbers, the search strategy did not include terms for parenting stress which may have led to under-identification of additional papers on moderators and mediators effects of parenting stress on the association between parenting practices and externalizing behaviors (considering the wealth of research on high levels of parenting stress in autistic parents, e.g., Costa et al., 2017; Dieleman et al., 2017; Frantz et al., 2018) which may be an important explicit focus in future reviews.

### Clinical Implications and Conclusion

Based on the findings of this review, fostering mindful parenting practices and reducing parenting practices associated with higher levels of externalizing behavior [control dimension (i.e., negative control, psychological control, overreactivity) and authoritarian parenting styles (low warmth, high control)], among parents of autistic children has valuable potential for supports for parents and their children. Review findings reinforce that parenting is a complex concept with varied definitions and measured with a range measures, making it difficult to compare, contrast, synthesise, or replicate results. Therefore, using consistent definitions in the field along with measures specific to this population is highlighted as an important area for future research. For example, the Aberrant Behavior Scale—Irritability subscale (Aman et al., 1985) has been a gold standard measure for measuring behavioral problems, specifically—irritability, as well as broadly—tantrums, verbal meltdowns, negative mood, and self-harm in autistic children (Stoddard et al., 2020), as well as medical interventions for behavioral problems in autism (see review by Persico et al., 2021). Given that parents of autistic children experience poorer psychological health as compared to other parents (see review by Enea & Rusu, 2020) and may be at increased risk of developing mental health challenges (Beer et al., 2013), which may likely affect parenting practices and subsequently child externalizing behaviors, it is important to identify data specific to this community. Therefore, for clinical purposes, it would be important to use autism-specific measures, as using measures only validated for the neurotypical populations may impact findings by not accurately capturing data related to the autistic community, therefore, likely impacting interventions and supports for this community. Further research into parenting practices and child externalizing behaviors and the potential effects of parenting stress on this association is imperative to inform the development of supports for the autism community by targeting modifiable variables, to help increase overall well-being of both parents and their children.

### Supplementary Information

Below is the link to the electronic supplementary material.

Supplementary file1 (DOCX 53 KB)

## References

[CR107] Achenbach, T. M. (1991). Manual for the Child Behavior Checklist 4–18 and 1991 profile. Burlington, VT: University of Vermont Department of Psychiatry.

[CR1] Adams D, Paynter J, Clark M, Roberts J, Keen D (2019). The developmental behaviour checklist (DBC) profile in young children on the autism spectrum: The impact of child and family factors. Journal of Autism and Developmental Disorders.

[CR4] Aguirre-Dávila E, Morales-Castillo M, Moreno-Vásquez M (2023). Parenting, autonomy and academic achievement in the adolescence. Journal of Family Studies.

[CR2] Aman, M. G., Mcdougle, C. J., Scahill, L., Handen, B., Arnold, L. E., Johnson, C., ... & Research Units on Pediatric Psychopharmacology Autism Network. (2009). Medication and parent training in children with pervasive developmental disorders and serious behavior problems: Results from a randomized clinical trial. *Journal of the American Academy of Child and Adolescent Psychiatry**, **48*(12), 1143–1154. 10.1097/CHI.0b013e3181bfd669 10.1097/CHI.0b013e3181bfd669 PMC3142923 19858761

[CR3] Aman, M. G., Singh, N. N., Stewart, A. W., & Field, C. (1985). Psychometric characteristics of the aberrant behavior checklist. *American Journal of Mental Deficiency**, **89*(5), 492–502. https://europepmc.org/article/med/3158201 3158201

[CR113] Aman M, Tassé MG (1996). The Nisonger CBRF: A child behavior rating form for children with developmental disabilities. Research in Developmental Disabilities.

[CR5] Arnold DS, O’Leary SG, Wolff LS, Acker MM (1993). The Parenting Scale: A measure of dysfunctional parenting in discipline situations. Psychological Assessment.

[CR6] Austin JK, Dunn DW, Johnson CS, Perkins SM (2004). Behavioral issues involving children and adolescents with epilepsy and the impact of their families: Recent research data. Epilepsy and Behavior.

[CR7] Aydin A (2022). Examining the mediating role of mindful parenting: A study on the relationship between parental emotion regulation difficulties and problem behaviors of children with ASD. Journal of Autism and Developmental Disorders.

[CR8] Bader SH, Barry TD (2014). A longitudinal examination of the relation between parental expressed emotion and externalizing behaviors in children and adolescents with autism spectrum disorder. Journal of Autism and Developmental Disorders.

[CR9] Bader SH, Barry TD, Hann JAH (2015). The relation between parental expressed emotion and externalizing behaviors in children and adolescents with an autism spectrum disorder. Focus on Autism and Other Developmental Disabilities.

[CR10] Badri M, Al Qubaisi A, Al Rashedi A, Yang G (2014). The causal relationship between parental involvement and children's behavioural adjustment to KG-1 schooling. International Journal of Child Care and Education Policy.

[CR11] Barber BK (1996). Parental psychological control: Revisiting a neglected construct. Child Development.

[CR12] Barroso NE, Mendez L, Graziano PA, Bagner DM (2018). Parenting stress through the lens of different clinical groups: A systematic review and meta-analysis. Journal of Abnormal Child Psychology.

[CR13] Baumrind D (1966). Effects of authoritative parental control on child behavior. Child Development.

[CR14] Bearss, K., Johnson, C., Smith, T., Lecavalier, L., Swiezy, N., Aman, M., ... & Scahill, L. (2015). Effect of parent training vs parent education on behavioral problems in children with autism spectrum disorder: A randomized clinical trial. *JAMA**, **313*(15), 1524–1533. 10.1001/jama.2015.3150 10.1001/jama.2015.3150 PMC9078140 25898050

[CR15] Beer M, Ward L, Moar K (2013). The relationship between mindful parenting and distress in parents of children with an autism spectrum disorder. Mindfulness.

[CR16] Benito, K. G., Caporino, N. E., Frank, H. E., Ramanujam, K., Garcia, A., Freeman, J., ... & Storch, E. A. (2015). Development of the pediatric accommodation scale: Reliability and validity of clinician- and parent-report measures. *Journal of Anxiety Disorders**, **29*, 14–24. 10.1016/j.janxdis.2014.10.004 10.1016/j.janxdis.2014.10.004 25481401

[CR17] Berliner SE, Moskowitz LJ, Braconnier M, Chaplin WF (2020). The role of parental attributions and discipline in predicting child problem behavior in preschoolers with and without autism spectrum disorder. Journal of Developmental and Physical Disabilities.

[CR18] Bitsika V, Sharpley CF (2019). Effects of diagnostic severity upon sex differences in behavioural profiles of young males and females with autism spectrum disorder. Journal of Autism and Developmental Disorders.

[CR19] Bögels SM, Hellemans J, van Deursen S, Römer M, van der Meulen R (2014). Mindful parenting in mental health care: Effects on parental and child psychopathology, parental stress, parenting, coparenting, and marital functioning. Mindfulness.

[CR20] Bögels SM, Lehtonen A, Restifo K (2010). Mindful parenting in mental health care. Mindfulness.

[CR21] Boonen H, Maljaars J, Lambrechts G, Zink I, Van Leeuwen K, Noens I (2014). Behavior problems among school-aged children with autism spectrum disorder: Associations with children's communication difficulties and parenting behaviors. Research in Autism Spectrum Disorders.

[CR23] Brinkman AH, Barry TD, Lindsey RA (2022). The relation of parental expressed emotion, parental affiliate stigma, and typically-developing sibling internalizing behavior in families with a child with ASD. Journal of Autism and Developmental Disorders.

[CR24] Burgdorf V, Szabó M, Abbott MJ (2019). The effect of mindfulness interventions for parents on parenting stress and youth psychological outcomes: A systematic review and meta-analysis. Frontiers in Psychology.

[CR25] Calders F, Bijttebier P, Bosmans G, Ceulemans E, Colpin H, Goossens L, Van Den Noortgate W, Verschueren K, Van Leeuwen K (2019). Investigating the interplay between parenting dimensions and styles, and the association with adolescent outcomes. European Child and Adolescent Psychiatry.

[CR27] Cheung RYM, Leung SSW, Mak WWS (2019). Role of mindful parenting, affiliate stigma, and parents’ well-being in the behavioural adjustment of children with autism spectrum disorder: Testing parenting stress as a mediator. Mindfulness.

[CR112] Chowdhury M, Aman MG (2016). Factor structure and psychometric properties of the revised Home Situations Questionnaire for autism spectrum disorder: The Home Situations Questionnaire-Autism Spectrum Disorder. Autism.

[CR28] Clauser P, Ding Y, Chen EC, Cho S-J, Wang C, Hwang J (2021). Parenting styles, parenting stress, and behavioural outcomes in children with autism. School Psychology International.

[CR29] Costa AP, Steffgen G, Ferring D (2017). Contributors to well-being and stress in parents of children with autism spectrum disorder. Research in Autism Spectrum Disorders.

[CR116] Covidence. (2022). Covidence Systematic Review Software, Veritas Health Innovation, Melbourne, Australia. https://www.covidence.org

[CR31] Crane L, Adams F, Harper G, Welch J, Pellicano E (2019). ‘Something needs to change’: Mental health experiences of young autistic adults in England. Autism: the International Journal of Research and Practice.

[CR33] Davies J, Glinn L, Osborne LA, Reed P (2022). Exploratory study of parenting differences for autism spectrum disorder and attachment disorder. Journal of Autism and Developmental Disorders.

[CR32] Davis NO, Carter AS (2008). Parenting stress in mothers and fathers of toddlers with autism spectrum disorders: Associations with child characteristics. Journal of Autism and Developmental Disorders.

[CR34] De Bruin EI, Blom R, Smit FM, Van Steensel FJ, Bögels SM (2015). MYmind: Mindfulness training for youngsters with autism spectrum disorders and their parents. Autism.

[CR35] De Clercq LE, Dieleman LM, van der Kaap-Deeder J, Soenens B, Prinzie P, De Pauw SSW (2021). Negative controlling parenting and child personality as modifiers of psychosocial development in youth with autism spectrum disorder: A 9-year longitudinal study at the level of within-person change. Journal of Autism and Developmental Disorders.

[CR37] De Clercq LE, Van der Kaap-Deeder J, Dieleman LM, Soenens B, Prinzie P, De Pauw SSW (2019). Parenting and psychosocial development in youth with and without autism spectrum disorder, cerebral palsy, and Down Syndrome: A cross-disability comparison. Advances in Neurodevelopmental Disorders: Multidisciplinary Research and Practice across the Lifespan.

[CR38] Dieleman LM, De Pauw SSW, Soenens B, Beyers W, Prinzie P (2017). Examining bidirectional relationships between parenting and child maladjustment in youth with autism spectrum disorder: A 9-year longitudinal study. Development and Psychopathology.

[CR39] Dieleman LM, De Pauw SSW, Soenens B, Mabbe E, Campbell R, Prinzie P (2018). Relations between problem behaviors, perceived symptom severity and parenting in adolescents and emerging adults with ASD: The mediating role of parental psychological need frustration. Research in Developmental Disabilities.

[CR40] Dieleman LM, Soenens B, Vansteenkiste M, Prinzie P, Laporte N, De Pauw SSW (2019). Daily sources of autonomy-supportive and controlling parenting in mothers of children with ASD: The role of child behaviour and mothers' psychological needs. Journal of Autism and Developmental Disorders.

[CR41] Duncan, L. G. (2007). Assessment of mindful parenting among parents of early adolescents: Development and validation of the Interpersonal Mindfulness in Parenting scale. Unpublished Dissertation.

[CR42] Emerson, E. (2001). *Challenging behaviour: Analysis and intervention in people with severe intellectual disabilities* (2^nd^ ed.). Cambridge University Press.

[CR43] Enea V, Rusu DM (2020). Raising a child with autism spectrum disorder: A systematic review of the literature investigating parenting stress. Journal of Mental Health Research in Intellectual Disabilities.

[CR108] Eyberg SM, Ross AW (1978). Assessment of child behavior problems: The validation of a new inventory. Journal of Clinical Child & Adolescent Psychology.

[CR44] Frantz R, Hansen SG, Machalicek W (2018). Interventions to promote well-being in parents of children with autism: A systematic review. Journal of Autism and Developmental Disorders.

[CR45] Galejs I, Pease D (1986). Parenting beliefs and locus of control orientation. The Journal of Psychology.

[CR47] Gerard, A. B. (1994). *Parent–child relationship inventory (PCRI)*. Western Psychological Services.

[CR115] Goodman M (1997). The Strengths and Difficulties Questionnaire: A research note. Journal of Child Psychology and Psychiatry.

[CR48] Gördesli, A. M., Arslan, R., Çek, F., Aydın Sünbül, Z., & Malkoç, A. (2018). The psychometric properties of the mindfulness in parenting questionnaire in Turkish sample. *European Journal of Education Studies, 5*(5), 175–188. https://files.eric.ed.gov/fulltext/ED591948.pdf

[CR49] Greenlee JL, Piro-Gambetti B, Putney J, Papp LM, Hartley SL (2022). Marital satisfaction, parenting styles, and child outcomes in families of autistic children. Family Process.

[CR50] Grolnick WS, Ryan RM, Deci EL (1991). Inner resources for school achievement: Motivational mediators of children's perceptions of their parents. Journal of Educational Psychology.

[CR52] Higgins JM, Arnold SRC, Weise J, Pellicano E, Trollor JN (2021). Defining autistic burnout through experts by lived experience: Grounded Delphi method investigating #autisticburnout. Autism.

[CR53] Jackson, A. P., & Choi, J. (2018). Parenting stress, harsh parenting, and children’s behaviour. *Journal of Family Medicine and Community Health, 5*(3), 10. https://www.researchgate.net/profile/Aurora-Jackson/publication/328137098_Parenting_Stress_Harsh_Parenting_and_Children's_Behavior/links/5bbfb58a92851c88fd651649/Parenting-Stress-Harsh-Parenting-and-Childrens-Behavior.pdf

[CR54] Jones L, Hastings RP, Totsika V, Keane L, Rhule N (2014). Child behaviour problems and parental well-being in families of children with autism: The mediating role of mindfulness and acceptance. American Journal on Intellectual and Developmental Disabilities.

[CR55] Kabat-Zinn, M., & Kabat-Zinn, J. (1997). *Everyday blessings: The inner work of mindful parenting*. Hyperion.

[CR56] Kerr DCR, Reinke WM, Eddy JM (2013). Trajectories of depressive symptoms and externalizing behaviors across adolescence: Associations with histories of suicide attempt and ideation in early adulthood. Suicide and Life-Threatening Behavior.

[CR64] La Buissonnière Ariza V, Schneider SC, Cepeda SL, Wood JJ, Kendall PC, Small BJ, Wood KS, Kerns C, Saxena K, Storch EA (2021). Predictors of suicidal thoughts in children with autism spectrum disorder and anxiety or obsessive–compulsive disorder: The unique contribution of externalizing behaviors. Child Psychiatry and Human Development.

[CR57] Lambrechts G, Van Leeuwen K, Boonen H, Maes B, Noens I (2011). Parenting behaviour among parents of children with autism spectrum disorder. Research in Autism Spectrum Disorders.

[CR65] Law, M., Stewart, C., Pollock, N., Letts, L., Bosch, J., & Westmorland, M. (1998). *McMaster critical review form-Quantitative studies*. McMaster University Occupational Therapy Evidence-Based Practice Research Group.

[CR58] Lebowitz ER, Panza KE, Su J, Bloch MH (2012). Family accommodation in obsessive–compulsive disorder. Expert Review of Neurotherapeutics.

[CR59] Lindsey RA, Saltness SR, Lau AF, Barry TD (2020). A longitudinal examination of interactions between autism symptom severity and parenting behaviors in predicting change in child behavior problems. Research in Autism Spectrum Disorders.

[CR60] Locke LM, Prinz RJ (2002). Measurement of parental discipline and nurturance. Clinical Psychology Review.

[CR61] Lucyshyn JM, Irvin LK, Blumberg ER, Laverty R, Horner RH, Sprague JR (2004). Validating the construct of coercion in family routines: Expanding the unit of analysis in behavioral assessment with families of children with developmental disabilities. Research and Practice for Persons with Severe Disabilities.

[CR62] Madarevic M, van Esch L, Lambrechts G, Ceulemans E, Van Leeuwen K, Noens I (2022). Parenting behaviors among mothers of pre-schoolers on the autism spectrum: Associations with parenting stress and children’s externalizing behaviour problems. Research in Autism Spectrum Disorders.

[CR63] Maljaars J, Boonen H, Lambrechts G, Van Leeuwen K, Noens I (2014). Maternal parenting behaviour and child behaviour problems in families of children and adolescents with autism spectrum disorder. Journal of Autism and Developmental Disorders.

[CR66] Marchand-Reilly JF (2012). The role of fathers’ depressive symptoms and lax and over-reactive discipline in children’s externalizing and internalizing behaviors. Journal of Adult Development.

[CR110] Mazefsky SM, Day TN (2018). Development of the emotion dysregulation inventory: A PROMIS®ing method for creating sensitive and unbiased questionnaires for autism spectrum disorder. Journal of Autism and Developmental Disorders.

[CR67] McRae EM, Stoppelbein L, O’Kelley SE, Fite P, Greening L (2018). Predicting internalizing and externalizing symptoms in children with ASD: Evaluation of a contextual model of parental factors. Journal of Autism and Developmental Disorders.

[CR68] McRae EM, Stoppelbein L, O’Kelley SE, Fite P, Greening L (2019). Predicting child behaviour: A comparative analysis between autism spectrum disorder and attention deficit/hyperactivity disorder. Journal of Child and Family Studies.

[CR114] Merrell, K. W. (2002). PKBS-2. Preschool and Kindergarten Behavior Scales. Austin, TX: PRO-ED.

[CR69] Mills AS, Tablon-Modica P, Mazefksy CA, Weiss JA (2022). Emotion dysregulation in children with autism: A multimethod investigation of the role of child and parent factors. Research in Autism Spectrum Disorders.

[CR70] Monk, R. (2022). *Autism terminology guidance from the Autistic community of Aotearoa New Zealand: A living resource created by Autistic people with the support of Autism New Zealand*. Autism New Zealand. https://autismnz.org.nz/resources/autism-new-zealand-terminology-guide/

[CR73] O’Nions E, Ceulemans E, Happé F, Benson P, Evers K, Noens I (2020). Parenting strategies used by parents of children with ASD: Differential links with child problem behaviour. Journal of Autism and Developmental Disorders.

[CR109] O'Nions SM, Christie AW (2014). Development of the ‘Extreme Demand Avoidance Questionnaire’(EDA‐Q): preliminary observations on a trait measure for Pathological Demand Avoidance. Journal of Child Psychology and Psychiatry.

[CR72] O’Nions E, Happé F, Evers K, Boonen H, Noens I (2018). How do parents manage irritability, challenging behaviors, non-compliance and anxiety in children with autism spectrum disorders? A meta-synthesis. Journal of Autism and Developmental Disorders.

[CR75] Osborne LA, McHugh L, Saunders J, Reed P (2008). The effect of parenting behaviors on subsequent child behaviour problems in autistic spectrum conditions. Research in Autism Spectrum Disorders.

[CR76] Ozsivadjian A, Hollocks MJ, Magiati I, Happé F, Baird G, Absoud M (2021). Is cognitive inflexibility a missing link? The role of cognitive inflexibility, alexithymia and intolerance of uncertainty in externalizing and internalizing behaviors in young people with autism spectrum disorder. Journal of Child Psychology and Psychiatry.

[CR77] Page, M. J., McKenzie, J. E., Bossuyt, P. M., Boutron, I., Hoffmann, T. C., Mulrow, C. D., Shamseer, L., Tetzlaff, J. M., Akl, E. A., Brennan, S. E., Chou, R., Glanville, J., Grimshaw, J. M., Hróbjartsson, A., Lalu, M. M., Li, T., Loder, E. W., Mayo-Wilson, E., McDonald, S., … Moher, D. (2021). The PRISMA 2020 statement: An updated guideline for reporting systematic reviews. *Journal of Clinical Epidemiology, 134*, 178–189. 10.1016/j.jclinepi.2021.03.001 10.1016/j.jclinepi.2021.03.001 33789819

[CR78] Park JL, Johnston C, Colalillo S, Williamson D (2018). Parents’ attributions for negative and positive child behavior in relation to parenting and child problems. Journal of Clinical Child and Adolescent Psychology.

[CR79] Pedro MF, Carapito E, Ribeiro T (2015). Parenting Styles and Dimensions Questionnaire—The Portuguese self-report version. Psicologia, Reflexão e Crítica.

[CR80] Pinquart, M. (2017). Associations of parenting dimensions and styles with externalizing problems of children and adolescents: An updated meta-analysis. *Developmental Psychology, 53*(5), 873–932. https://oce-ovid-com.libraryproxy.griffith.edu.au/article/00063061-201705000-00006/HTML 10.1037/dev0000295 28459276

[CR81] Portes J, Vieira M, de Souza CD, Kaszubowski E (2020). Parental styles and coparenting in families with children with autism: Cluster analysis of children’s behaviour. Estudos De Psicologia (campinas).

[CR82] Rahman, P. A., & Jermadi S. H. (2021). Parental stress and parenting styles in managing autistic children with behaviour problems. *Malaysian Journal of Medicine and Health Sciences, 17*, 84–91. https://medic.upm.edu.my/upload/dokumen/2021060913532012_2020_1168.pdf

[CR83] Raulston TJ, Kosty D, McIntyre LL (2021). Mindful parenting, caregiver distress, and conduct problems in children with autism. American Journal on Intellectual and Developmental Disabilities.

[CR106] Reynolds, C. R., & Kamphaus, R. W. (2004). Behavior Assessment System for Children . Bloomington, MN: Pearson Assessments.

[CR84] Ridderinkhof A, de Bruin EI, Blom R, Bögels SM (2018). Mindfulness-based program for children with autism spectrum disorder and their parents: Direct and long-term improvements. Mindfulness.

[CR85] Robertson AE, Simmons DR (2015). The sensory experiences of adults with autism spectrum disorder: A qualitative analysis. Perception.

[CR86] Robinson CC, Mandleco B, Olsen SF, Hart CH (2001). The parenting styles and dimensions questionnaire (PSDQ). Handbook of Family Measurement Techniques.

[CR87] Schiltz, H. K., McVey, A. J., Magnus, B., Dolan, B. K., Willar, K. S., Pleiss, S., ..., Van Hecke, A. V. (2018). Examining the links between challenging behaviors in youth with ASD and parental stress, mental health, and involvement: Applying an adaptation of the family stress model to families of youth with ASD. *Journal of Autism and Developmental Disorders, 48*, 1169–1180. 10.1007/s10803-017-3446-0 10.1007/s10803-017-3446-0 PMC10321230 29275509

[CR88] Shawler PM, Sullivan MA (2017). Parental stress, discipline strategies, and child behavior problems in families with young children with autism spectrum disorders. Focus on Autism and Other Developmental Disabilities.

[CR89] Shelton KK, Frick PJ, Wootton J (1996). Assessment of parenting practices in families of elementary school-age children. Journal of Clinical Child Psychology.

[CR111] Shields SM, Cicchetti TN (1997). Emotion regulation among school-age children: The development and validation of a new criterion Q-sort scale. Developmental Psychology.

[CR90] Singh NN, Lancioni GE, Winton ASW, Fisher BC, Wahler RG, Mcaleavey K, Singh J, Sabaawi M (2006). Mindful parenting decreases aggression, noncompliance, and self-injury in children with autism. Journal of Emotional and Behavioral Disorders.

[CR91] Smith CG, Jones EJH, Wass SV, Pasco G, Johnson MH, Charman T, Wan MW (2021). Infant effortful control mediates relations between nondirective parenting and internalizing-related child behaviors in an autism-enriched infant cohort. Journal of Autism and Developmental Disorders.

[CR92] Soto-Sanz V, Castellví P, Piqueras JA, Rodríguez-Marín J, Rodríguez-Jiménez T, Miranda-Mendizábal A, Parés-Badell O, Almenara J, Alonso I, Blasco MJ, Cebrià A, Gabilondo A, Gili M, Lagares C, Roca M, Alonso J (2019). Internalizing and externalizing symptoms and suicidal behaviour in young people: A systematic review and meta-analysis of longitudinal studies. Acta Psychiatrica Scandinavica.

[CR93] Sterling-Turner HE, Jordan SS (2007). Interventions addressing transition difficulties for individuals with autism. Psychology in the Schools.

[CR94] Stoddard J, Zik J, Mazefsky CA, DeChant B, Gabriels R (2020). The internal structure of the aberrant behavior checklist irritability subscale: Implications for studies of irritability in treatment-seeking youth with autism spectrum disorders. Behavior Therapy.

[CR95] Storch EA, Zavrou S, Collier AB, Ung D, Arnold EB, Mutch PJ, Lewin AB, Murphy TK (2015). Preliminary study of family accommodation in youth with autism spectrum disorders and anxiety: Incidence, clinical correlates, and behavioural treatment response. Journal of Anxiety Disorders.

[CR96] Strömberg M, Liman L, Bang P, Igelström K (2022). Experiences of sensory overload and communication barriers by autistic adults in health care settings. Autism in Adulthood.

[CR97] Tellegen CL, Sanders MR (2014). A randomized controlled trial evaluating a brief parenting program with children with autism spectrum disorders. Journal of Consulting and Clinical Psychology.

[CR99] Ueda MM, Ding Y, Blumberg F, Zhang C, Yu Q, Lantier K (2020). Maternal parenting style in relation to parenting stress and behavioural Outcomes in Japanese children with and without autism. Journal of Developmental and Physical Disabilities.

[CR100] Van Leeuwen, K. G., & Noens, I. (2013). Parental behavior scale for autism spectrum disorders. Unpublished Document, KU Leuven.

[CR101] Van Leeuwen KG, Vermulst AA (2004). Some psychometric properties of the Ghent parental behavior scale1. European Journal of Psychological Assessment.

[CR102] Ventola P, Lei J, Paisley C, Lebowitz E, Silverman W (2017). Parenting a child with ASD: Comparison of parenting style between ASD, anxiety, and typical development. Journal of Autism and Developmental Disorders.

[CR103] Whittingham K, Sofronoff K, Sheffield J, Sanders MR (2009). Stepping stones triple P: An RCT of a parenting program with parents of a child diagnosed with an autism spectrum disorder. Journal of Abnormal Child Psychology: An Official Publication of the International Society for Research in Child and Adolescent Psychopathology.

[CR104] Wiedemann, G., Rayki, O., Feinstein, E., & Hahlweg, K. (2002). The Family Questionnaire: Development and validation of a new self-report scale for assessing expressed emotion. *Psychiatry Research**, **109*(3), 265–279. https://www.researchgate.net/profile/Kurt-Hahlweg/publication/11408725_The_Family_Questionnaire_Development_and_validation_of_a_new_self-report_scale_for_assessing_expressed_emotion/links/5f9d38ab299bf1b53e547971/The-Family-Questionnaire-Development-and-validation-of-a-new-self-report-scale-for-assessing-expressed-emotion.pdf 10.1016/s0165-1781(02)00023-9 11959363

[CR105] Williams, K., & Roberts, J. (2018). *Understanding autism: The essential guide for parents* (Vol. 3). Exisle Publishing. https://books.google.com.au/books?hl=en&lr=&id=0mJUDwAAQBAJ&oi=fnd&pg=PP1&dq=Williams,+K.,+%26+Roberts,+J.+(2018).+Understanding+autism:+The+essential+guide+for+parents+(Vol.+3).+Exisle+Publishing&ots=nAz1HDpCjJ&sig=E3AUPzrHDIRIzBFeN6P17tSTcUk&redir_esc=y#v=onepage&q&f=false

