# Supplementary material for: Parenting Practices and Externalizing Behaviors in Autistic Children: A Systematic Literature Review
Source: Clin Child Fam Psychol Rev. 2024 Feb 26;27(1):235–56. doi: 10.1007/s10567-024-00467-6 (PMC10920481; doi:10.1007/s10567-024-00467-6)
Supplement: Supplementary file 1 — Supplementary file1 (DOCX 53 KB) [file 10567_2024_467_MOESM1_ESM.docx]

**Supplementary Material**

**Table 2.**

*Search Terms for the Systematic Review.*

| Key Variables | Search Terms |
| --- | --- |
| Target population: Child | autis* OR asd OR asperger* OR "Pervasive Developmental Disorder" OR pdd OR pdd-nos  AND |
| Target population: Parent & Parenting practices | parent* OR parenting OR “parent* behavio*” OR “parent* practice*” OR “parent* style"  OR  "parent over reactivity"  OR  "parent over-reactivity"  OR  "parent* warmth"  OR  "parent* behav*"  OR  "parent* style"  OR  "limit setting"  OR  limit-setting  OR  accommodation OR  forbearance  OR  structure  OR  "parent* control"  AND |
| Child behaviors | behavi* OR “behavio* problem” OR “challenging behavi*” OR “behavi* of concern” OR noncompl* OR non-compl* OR irritab* OR “demand avoid*” OR “avoids demand*” OR “emotion* dysregulation” OR “emotion* regulation” OR “emotion* respons*” OR aggression OR “self injur*” OR self-injur* OR “maladaptive behavio*” OR “problem behavio*” OR “aberrant behavio*” OR self-harm OR “aggressive behavio*” OR “disruptive behavio*” OR “destructive behavio*” OR “destruction of property” OR “behavio* difficulties” OR “externali* behavio*” |

**Table 3.**

*Parenting Classification Scheme for Measures Used by Included Studies.*

| Measure | Reference | Parenting Classification Scheme for Review |
| --- | --- | --- |
| Alabama Parenting Questionnaire (APQ, split into positive [subscales - Parental involvement and Positive parenting] and Negative parenting composite scores [Poor monitoring/supervision, Inconsistent discipline, and Corporal punishment]) (*n* = 5). | (Shelton et al., 1996) | Parenting practices – Warmth and Control dimensions |
| Bangor Mindful Parenting scale (BMPS Total score) (*n* = 3). | (Jones et al., 2014) | Mindful parenting practices |
| Family Questionnaire subscales (FQ subscales Parental criticism and Emotional overinvolvement) (*n* = 1). | (Wiedemann et al., 2002) | Parenting practices – Control dimension |
| Interpersonal Mindfulness in Parenting Scale (IM-P Total score) (*n* = 1). | (Duncan, 2007) | Mindful parenting practices |
| Mindfulness in Parenting Questionnaire – Turkish adaptation (MIPQ Total score) (*n* = 1). | (Gördesli et al., 2018) | Mindful parenting practices |
| Paediatric Accommodation Scale (PAS subscales Frequency, Parent Impact, and Child Impact) (*n* = 1). | (Benito et al., 2015) | Parenting practices – Parenting Strategies to Support Externalizing Behaviors |
| Parental Behavior Scale subscale or composite scores (PBS composite scores [Positive parenting – Positive parenting subscale + Rules subscale; Negative control – Discipline and Harsh punishment]) (*n* = 3). | (Van Leeuwen & Vermulst, 2004) | Parenting practices – Warmth and Control dimensions |
| The Parental Behavior Scale-ASD subscale or composite scores (PBS-A composite scores, Positive parenting – Positive parenting, Material rewarding and Rules); Negative control (Discipline and Harsh punishment); Autism adapted parenting (Stimulating Development and Adapting the Environment) (*n* = 3). | (Van Leeuwen & Noens, 2013) | Parenting practices – Warmth and Control dimensions. Parenting practices – Parenting Strategies to Support Externalizing Behaviors |
| Parent–Child Relationship Inventory (PCRI) (*n* = 2). | (Gerard, 1994) | Parenting practices – Warmth and Control dimensions |
| Psychological Control Scale (PCS total score) (*n* = 3). | (Barber, 1996) | Parenting practices – Control dimension |
| Parenting Scale (PS Total score - Laxness + Overreactivity subscales, *n* = 1) and (Laxness and Overreactivity subscales, *n* = 2). | (Arnold et al., 1993) | Parenting practices – Control dimension |
| Autonomy Support Scale of the Perceptions of Parents Scale (POPS Autonomy Support subscale) (*n* = 3). | (Grolnick et al., 1991) | Parenting practices – Warmth dimension |
| Parent Report of Parenting Behavior Inventories (PRPBI Total scores and Acceptance, Psychological control, and Firmness subscales) (*n* = 1). | (Galejs & Pease, 1986) | Parenting practices – Warmth and Control dimensions |
| The Parenting Styles and Dimensions Questionnaire (PSDQ) (*n* = 4). | (Robinson et al., 2001) | Parenting Practices - Parenting Styles (Authoritarian, Authoritative, Permissive) |
| The Parenting Styles and Dimensions Questionnaire (PSDQ) Portuguese adaptation, mother and father versions  (*n* = 1) | (Pedro et al., 2015) | Parenting Practices - Parenting Styles (Authoritarian, Authoritative, Permissive) |
| Parenting Strategies Questionnaire (PSQ (*n* = 1) | (O’Nions et al., 2018) | Parenting practices – Parenting Strategies to Support Externalizing Behaviors |

**Table 4**

*Risk of Bias*

| Included Papers | Risk of bias | | | | | | | | | | |
| --- | --- | --- | --- | --- | --- | --- | --- | --- | --- | --- | --- |
|  | Study Purpose | Sample details | Sample size justification | Sample bias - nil | Validity | Reliability | Statistically significant | Effect size reported | Ethics and informed consent | Total score used to measure child externalizing behaviors | Conclusion and implications |
| Aydin, 2022 | Y | N | N | Y | N | Y | Y | Y | Y | Y | Y |
| Bader & Barry, 2014 | Y | Y | N | Y | N | Y | Y | Y | Y | N | Y |
| Bader et al., 2015 | Y | Y | N | Y | N | Y | Y | Y | Y | N | Y |
| Beer et al., 2013 | Y | Y | N | Y | N | Y | Y | Y | Y | Y | Y |
| Berliner et al., 2020 | Y | Y | Y | Y | N | Y | Y | Y | Y | N | Y |
| Boonen et al., 2014 | Y | Y | Y | Y | Y | Y | Y | Y | Y | N | Y |
| Brinkman et al., 2022 | Y | Y | N | Y | N | Y | Y | Y | Y | N | Y |
| Cheung et al., 2019 | Y | Y | Y | Y | N | Y | Y | Y | Y | Y | Y |
| Clauser et al., 2021 | Y | Y | Y | Y | N | Y | Y | Y | Y | N | Y |
| Davies et al., 2022 | Y | Y | Y | Y | N | Y | Y | Y | Y | Y | Y |
| De Clercq et al., 2021 | Y | Y | Y | Y | N | Y | Y | Y | Y | N | Y |
| De Clercq et al., 2019 | Y | Y | Y | Y | N | Y | Y | Y | Y | N | Y |
| Dieleman et al., 2017 | Y | Y | Y | Y | N | Y | Y | Y | Y | N | Y |
| Dieleman et al., 2018 | Y | Y | Y | Y | N | Y | Y | Y | Y | N | Y |
| Dieleman et al., 2019 | Y | Y | Y | Y | N | Y | Y | Y | Y | N | Y |
| Greenlee et al., 2022~~0~~ | Ygree | Y | Y | Y | N | Y | Y | Y | Y | N | Y |
| Lindsey et al., 2020. | Y | Y | Y | Y | N | Y | Y | Y | Y | N | Y |
| Maljaars et al., 2014 | Y | N | N | Y | Y | Y | Y | Y | Y | N | Y |
| McRae et al., 2018 | Y | N | N | Y | N | Y | Y | Y | Y | N | Y |
| McRae et al., 2019 | Y | N | N | N | N | Y | Y | Y | Y | N | Y |
| Mills et al., 2022 | Y | Y | N | Y | N | Y | Y | Y | Y | N | Y |
| O’Nions et al., 2020 | Y | Y | N | Y | Y | Y | Y | Y | Y | N | Y |
| Osborne et al., 2008 | Y | N | N | Y | N | Y | Y | Y | N | Y | Y |
| Portes et al., 2020 | Y | N | N | Y | N | N | NA | N | Y | Y | Y |
| Rahman, & Jermadi, 2021. | Y | Y | N | Y | N | Y | Y | Y | Y | N | Y |
| Raulston et al., 2021 | Y | Y | Y | Y | N | Y | Y | Y | Y | N | Y |
| Shawler & Sullivan, 2017 | Y | Y | N | Y | N | Y | Y | Y | Y | N | Y |
| Storch et al., 2015 | Y | N | N | Y | N | Y | N | Y | Y | N | Y |
| Ueda et al., 2020 | Y | Y | N | Y | N | Y | Y | Y | Y | N | Y |
| Ventola et al., 2017 | Y | N | N | Y | N | Y | Y | Y | Y | N | Y |

**Table 5**

*Associations Between Parenting Practices (Parenting Styles) and Externalizing Behaviors.*

| **Reference** | **Design & Analyses** | **Association between child externalizing behaviors and parenting practices** | **Significance** | **Other outcomes (e.g., other parent and child variables - control, mediator or moderator variables, and parent mental health)** |
| --- | --- | --- | --- | --- |
| Clauser et al., 2021 | Cross-sectional. Pearson correlation analyses and hierarchical regression. | Overall model R^2^ = .62, *p* =.04  Permissive b=.11 (unique variance = .012) Authoritative b =.08 (unique variance = .006) Authoritarian b = .19 (unique variance = 0.036) R2 = .57 | ****p* < .001  ***p* < .01  **p* < .05 | Associations between parenting stress (PSI-SF) and parenting practices.  Parent mental health –  Parenting Stress Index (PSI) |
| Greenlee et al., 2022~~0~~ | Longitudinal. Paired samples t-test & Mediation analyses. | Mother  Time 2 parenting practices (authoritarian) and Time 3 child externalizing behaviors *r* = .31**  Time 2 parenting practices (authoritative) and Time 3 child externalizing behaviors *r* = .22*  Time 2 parenting practices (permissive) and Time 3 child externalizing behaviors *r* = .20*  Father  Time 2 parenting practices (authoritarian) and Time 3 child externalizing behaviors *r* = .47**  Time 2 parenting practices (authoritative) and Time 3 child externalizing behaviors *r* = -.30**  Time 2 parenting practices (permissive) and Time 3 child externalizing behaviors *r* = .40* | ***p* < .01  **p* < .05 | Autism characteristics.  Parent practices and marital relationship satisfaction (mediation effects). |
| Portes et al., 2020 | Cross-sectional. Hierarchical clustering technique. | Profile 1: High child externalizing behaviors (highest) and prosocial behaviors.  Mothers perceive their parenting styles as – permissive, followed by authoritative and negative levels of authoritarian. Fathers also perceived their parenting styles as permissive and authoritarian, followed by authoritative.  Profile 2: Borderline child externalizing behaviors and prosocial difficulties. Mothers perceive their parenting styles as – permissive, followed by authoritative and negative levels of authoritarian. Fathers perceived their parenting styles as authoritative, followed by permissive, and negative levels of authoritarian.  Profile 3: High prosocial behaviors and low child externalizing behaviors. Mothers showed lowest level of permissive parenting and negatives levels of authoritarian and authoritative styles. Fathers showed lowest levels of permissive parenting and authoritarian styles, with positive levels of authoritative parenting.  Profile 4: Limitations in prosocial behaviors and high child externalizing behaviors. Both mother and father perceived their parenting styles as lowest levels of authoritative styles and highest for authoritarian, whereas negative levels of permissive styles. | NA | Coparenting relationship |
| Rahman, & Jermadi, 2021 | Cross-sectional. Pearson’s chi-squared test and correlations. | Parenting practices (authoritarian) and externalizing behaviors (conduct problems) *r* = 0.12 non-significant  Parenting practices (authoritative) and externalizing behaviors (conduct problems) *r* = - 0.02 non-significant  Parenting practices (permissive) and externalizing behaviors (conduct problems) *r* = 0.12 non-significant  Parenting practices (authoritarian) and externalizing behaviors (hyperactive) *r* = .36***  Parenting practices (authoritative) and externalizing behaviors (hyperactive) *r* = - 0.04 non-significant.  Parenting practices (permissive) and externalizing behaviors (hyperactive) *r* = 0.33 non-significant | ****p* < .001  ***p* < .01  **p* < .05 | Parent mental health –  PSI |
| Ueda et al., 2020 | Cross-sectional. The Mann-Whitney U test (group differences), Independent samples t-test, and Spearman rank correlations. | Parenting practices (authoritative) and externalizing behaviors *r* = - .02 non-significant.  Parenting practices (authoritarian) and externalizing behaviors *r* = .35*.  Parenting practices (permissive) and externalizing behaviors *r* = .50**.  Regression analyses  Parenting styles did not have significant effects on child externalizing behaviors. | ****p* < .001  ***p* < .01  **p* < .05 | Autism characteristics.  Parent perceived social support.  Parent mental health –  PSI-SF |

**Table 6**

*Associations Between Parenting Practices (Across Dimensions of Warmth and Control and Strategies to Support Behaviours) and Externalizing Behaviors.*

| **Reference** | **Design & Analyses** | **Association between child externalizing behaviors and parenting practices** | **Significance values** | **Other outcomes (e.g., other parent and child variables - control, mediator or moderator variables, and parent mental health)** |
| --- | --- | --- | --- | --- |
| Bader & Barry, 2014 | Longitudinal. Correlations and hierarchical multiple regression (HMR) | Time 1 Parenting practices (positive) and child externalizing behaviors *r* = -.10 non-significant  Time 1 Parenting practices (negative) and Time 1 child externalizing behaviors *r* = .36*  Time 1 Parenting practices (positive) and Time 2 child externalizing behaviors *r* = -.02 non-significant  Time 1 Parenting practices (negative) and Time 2 child externalizing behaviors *r* = .39*** | **p* < .05  ***p* < .01  ****p* < .001 | Parent expressed emotion and children’s social behaviour questionnaire.  Parent mental health –  Parenting Stress Index (PSI) |
| Bader et al., 2015 | Cross-sectional. Zero-order correlations and HMR | Parenting practices (negative control) and child externalizing behaviors r = .34***  Parenting practices (positive) and child externalizing behaviors r = -.01 non-significant | **p* < .05  ***p* < .01  ****p* < .001 | HMR to analyse if parts of parent expressed emotion (criticism/hostility and emotional overinvolvement) as predictors of child externalizing  behaviors.  Parent mental health – PSI |
| Berliner et al., 2020 | Cross-sectional. Regression based path analysis and Independent t-tests | Regression model (3 predictors – child autism status, parent attributions of controllability and parent lax discipline)  *R^2^* = .46***  Parental lax discipline(*β* = .17, *p* = .015) contributed significantly to the model. | ****p* < .001 | Differences in parental attributions between parents of autistic children and neurotypical children. |
| Boonen et al., 2014 | Cross-sectional. Independent samples *T*-test and HMR | Parenting practices (negative control) and child externalizing behaviors r = .28**  Parenting practices (positive) and child externalizing behaviors r = .16* significant  Parenting practices (Autism adapted parenting) and child externalizing behaviors r = .10 non-significant. | **p* < .05  ***p* < .01 | Associations between child externalizing behaviors and child characteristics.  Child related predictors of externalizing behaviors.  Moderator effects of child (communication difficulties) and family variables (parenting practices). |
| Brinkman et al., 2022 | Cross-sectional. Correlations and HMR | Parenting practices (parental criticism) and child externalizing behaviors *p* = .63***  Parenting practices (emotional overinvolvement) and child externalizing behaviors *p* = .37*** (partial correlation) | ****p* < .001  ***p* < .01  **p* < .05 | Associations between child externalizing behaviors and parenting stress and parental affiliate stigma.  Parent mental health – DASS |
| Davies et al., 2022 | Cross-sectional. MANOVA | PCRI Limit setting and child externalizing behaviors *r* =  -.64***  PCRI Communication and child externalizing behaviors *r* = .19 non-significant  PCRI Satisfaction and child externalizing behaviors *r* = -.01 non-significant  PCRI Involvement and child externalizing behaviors *r* = -.02 non-significant | ****p* < .001  ***p* < .01  **p* < .05 | Group differences.  Impact of parent stress on child behaviors.  Parent mental health –  Parenting Stress Index (PSI) |
| De Clercq et al., 2021 | Longitudinal. Latent change models. | Time 1 parenting practices (negative control) and Time 1 child externalizing behaviors,  *r* = .27**  Time 2 parenting practices (negative control) and Time 1 child externalizing behaviors, *r* = .39***  Time 3 parenting practices (negative control) and Time 1 child externalizing behaviors, *r* = .40***  Time 2 parenting practices (negative control) and Time 2 child externalizing behaviors, *r* = .46***  Time 1 parenting practices (negative control) and Time 2 child externalizing behaviors, *r* = .25*  Time 3 parenting practices (negative control) and Time 2 child externalizing behaviors, *r* = .36*  Time 3 parenting practices (negative control) and Time 3 child externalizing behaviors, *r* = .34***  Time 2 parenting practices (negative control) and Time 3 child externalizing behaviors, *r* = .48***  Time 1 parenting practices (negative control) and Time 3 child externalizing behaviors, *r* = .22* | ****p* < .001  ***p* < .01  **p* < .05 | Other child variables – child personality, autism characteristics, and child psychosocial strength. |
| De Clercq et al., 2019 | Cross-sectional.  Bivariate correlation analyses, Kruskal-Wallis H tests, and Multi-group structural equation modeling (SEM). | Parenting practices (psychological control) and externalizing behaviors *r* = .23* | ****p* < .001  ***p* < .01  **p* < .05 | Child psychosocial strengths. |
| Dieleman et al., 2017 | Longitudinal. Cross-lagged modeling, Correlations. | Time 1 externalizing behaviors and parenting practices (negative control) *r* = .26**  Time 1 externalizing behaviors and parenting practices (positive) *r* = .07 non-significant  Time 1 externalizing behaviors and Time 2 parenting practices (negative control*) r* = .39***  Time 1 externalizing behaviors and Time 2 parenting practices (positive) *r* = .14 non-significant  Time 1 externalizing behaviors and Time 3 parenting practices (negative control*) r* = .39***  Time 1 externalizing behaviors and Time 3 parenting practices (positive*) r* = .20*  Time 2 externalizing behaviors parenting practices (negative control*) r* = .46***  Time 2 externalizing behaviors parenting practices (positive) *r* = .04 non-significant  Time 2 externalizing behaviors and Time 1 parenting practices (negative control*) r* = .25*  Time 2 externalizing behaviors and Time 1 parenting practices (positive) *r* = .05 non-significant  Time 2 externalizing behaviors and Time 3 parenting practices (negative control*) r* = .36***  Time 2 externalizing behaviors and Time 3 parenting practices (positive*) r* = .33**  Time 3 externalizing behaviors and parenting practices (negative control*) r* = .36***  Time 3 externalizing behaviors and parenting practices (positive) *r* = .13 non-significant  Time 3 externalizing behaviors and Time 1 parenting practices (negative control*) r* = .24*  Time 3 externalizing behaviors and Time 1 parenting practices (positive) *r* = .09 non-significant  Time 3 externalizing behaviors and Time 2 parenting practices (negative control*) r* = .48***  Time 3 externalizing behaviors and Time 2 parenting practices (positive) *r* = .05 non-significant  CROSS LAGGED MODEL RESULTS: Externalizing behaviors predicted a significant increase in parenting practices (negative control). Parenting practices (negative control) during adolescence (Time2) predicted an increase in externalizing behaviors (after 3 years). Externalizing behaviors at Time 2 predicted parenting practices (positive behaviors) after 3 years. | ****p* < .001  ***p* < .01  **p* < .05 | Autism characteristics. |
| Dieleman et al., 2018 | Cross-sectional. Correlations, SEM. | Child externalizing behaviors (aggression) and parenting practices (autonomy support) *r* = -.08 non-significant.  Child externalizing behaviors (aggression) and parenting practices (overreactivity) *r* = .04***  Child externalizing behaviors (aggression) and parenting practices (psychological control) *r* = .39***  Child externalizing behaviors (rule breaking) and parenting practices (autonomy support) *r* = -.03 non-significant  Child externalizing behaviors (rule breaking) and parenting practices (overreactivity) *r* = .27**  Child externalizing behaviors (rule breaking) and parenting practices (psychological control) *r* = .30** | ****p* < .001  ***p* < .01  **p* < .05 | Autism characteristics.  Parent mental health –  Parenting Stress Index (PSI) |
| Dieleman et al., 2019 | Cross-sectional. Multilevel SEM | Child externalizing behaviors and parenting practices (autonomy-supportive) *r* = -.09 non-significant  Child externalizing behaviors and parenting practices (controlling parenting) *r* = .51*** | ****p* < .001  ***p* < .01  **p* < .05 | Autism characteristics and child prosocial behaviors.  Parent Daily Psychological Need Satisfaction and Need  Frustration and Vitality  Parent mental health –  DASS |
|  |  |  |  |  |
| Lindsey et al., 2020 | Longitudinal. Multiple regression | Time 1 parenting practices (positive parenting) and Time 2 child externalizing behaviors *r* = -.19*  Time 1 parenting practices (negative parenting) and Time 2 child externalizing behaviors *r* = .42***  Negative parenting practices predicted unique variance in child externalizing behaviors, whereas positive parenting practices did not. | ****p* < .001  ***p* < .01  **p* < .05 | Autism characteristics.  Moderator effects of parenting practices |
|  |  |  |  |  |
| Maljaars et al., 2014 | Cross-sectional. MANOVA (group differences) and correlations. | Parenting practices (positive parenting) and externalizing behaviors *r* = non-significant  Parenting practices (harsh punishment) and externalizing behaviors *r* = .27*  Parenting practices (material rewarding) and externalizing behaviors *r* = non-significant  Parenting practices (rules) and externalizing behaviors *r* = .25*  Parenting practices (discipline) and externalizing behaviors r = .27*  Parenting practices (stimulating the development) and externalizing behaviors *r* = non-significant  Parenting practices (adapting the environment) and externalizing behaviors *r* = non-significant. | **p* < .007 after Bonferroni correction | NA |
| McRae et al., 2018 | Cross-sectional. Correlations, One-sample t-tests, and Path analysis. | Parenting practices (harsh/disengaged) and externalizing behaviors *r* = .38**  Parenting practices (warm/supportive) and externalizing behaviors *r* = non-significant  Path analysis  Parenting practices (harsh/disengaged) predicted externalizing behaviors *b* = .59**, such that more harsh/disengaged parenting practices predicted more child externalizing behaviors. | ***p* < .01  **p* < .05 | Child routines.  Parent mental health - The Hopkins Symptom Checklist- 25 (HSCL-25). |
| McRae et al., 2019 | Cross-sectional. Correlations, t-tests, and Path analysis. | Parenting practices (warm/supportive) and externalizing behaviors *r* = non-significant  Parenting practices (harsh/disengaged) and externalizing behaviors *r* = non-significant  Path analysis (Autism & ADHD groups included)  Harsh/disengaged parenting practices predicted child externalizing behaviors *b* = 0.34, *p* <.02 | ***p* < .01  **p* < .05 | Child routines.  Parent mental health - The Hopkins Symptom Checklist- 25 (HSCL-25). |
| O’Nions et al., 2020 | Cross-sectional. PCA with Varimax rotations (covariance among PSQ items), Correlations, & Regression analysis. | Parenting practices (accommodation) and externalizing behaviors (reactivity) *r* = .50***  Parenting practices (accommodation) and externalizing behaviors (demand specific non-compliance) *r* = 0.41***  Parenting practices (accommodation) and externalizing behaviors (extreme demand avoidance) *r* = .44***  Parenting practices (accommodation) and externalizing behaviors (socially inflexible) *r* = .53***  Parenting practices (reducing uncertainty) and externalizing behaviors (reactivity) *r* = .20**  Parenting practices (reducing uncertainty) and externalizing behaviors (demand specific non-compliance) *r* = .13 non-significant  Parenting practices (reducing uncertainty) and externalizing behaviors (extreme demand avoidance) *r* = .15*  Parenting practices (reducing uncertainty) and externalizing behaviors (socially inflexible) *r* = .22**  Parenting practices (reinforcement approaches) and externalizing behaviors (reactivity) *r* = .02 non-significant  Parenting practices (reinforcement approaches) and externalizing behaviors (demand specific non-compliance) *r* = -.03 non-significant  Parenting practices (reinforcement approaches) and externalizing behaviors (extreme demand avoidance) *r* = -.13*  Parenting practices (reinforcement approaches) and externalizing behaviors (socially inflexible) *r* = -.05 non-significant | ****p* < .001  ***p* < .01  **p* < .05 | Autism characteristics. |
| Osborne et al., 2008 | Longitudianl. Correlations, Time-lagged correlations, and Mediation analyses (parenting practices and parenting stress as mediator variables). | BASELINE  Parenting practices (involvement) and externalizing behaviors *r* = - 0.14 (non-significant)  Parenting practices (communication) and externalizing behaviors *r* = - 0.12 non-significant  Parenting practices (limit setting) and externalizing behaviors *r* = -.55***  Parenting practices (autonomy) and externalizing behaviors *r* = -0.15 non-significant  FOLLOW-UP  Parenting practices (involvement) and externalizing behaviors *r* = -.31***  Parenting practices (communication) and externalizing behaviors *r* = -0.13 non-significant  Parenting practices (limit setting) and externalizing behaviors *r* = -.42***  Parenting practices (autonomy) and externalizing behaviors *r* = 0.07 non-significant  Time-lagged correlations  Baseline parenting practices (limit setting) and externalizing behaviors at follow-up was statistically significant (xx) | ****p* < .001  ***p* < .01  **p* < .05 | Autism characteristics, cognitive abilities, adaptive functioning skills.  Parent-Child relationship inventory.  Parent mental health –  The Friedrich Short-Form of the QRS.  Mediating effects of parenting practices and parenting stress. |
| Shawler & Sullivan, 2017 | Cross-sectional. Correlations and Mediation analyses (parenting practices as mediator) | Parenting practices (total) and externalizing behaviors *r* = .26**  Parenting practices (laxness) and externalizing behaviors *r* = .13 non-significant  Parenting practices (overreactivity) and externalizing behaviors *r* = .26** | ****p* < .001  ***p* < .01  **p* < .05 | Autism characteristics.  Parent mental health –  PSI-SF |
| Storch et al., 2015 | Cross-sectional. Pearson’s correlations, and dependent and between groups *T-*tests. | Parenting practices (accommodation frequency) and externalizing behaviors r = .27 non-significant  Parenting practices (parent impact) and externalizing behaviors r = -.27 non-significant  Parenting practices (child impact) and externalizing behaviors r = -.24 non-significant | ***p* < .01  **p* < .05 | Child anxiety and comorbid disorders, anxiety symptoms, improvement scale, and social engagement abilities. |
| Ventola et al., 2017 | Cross-sectional. Partial correlations, ANOVA (group differences) and MANCOVA (differences between the 3 groups) | Parenting practices (psychological control) and externalizing behaviors *r* = .29* | ****p* < .001  ***p* < .01  **p* < .05 | Parent mental health –  Beck Anxiety Inventory and Beck Depression Inventory. |

**Table 7**

*Associations Between Parenting Practices (Mindful Parenting) and Externalizing Behaviors.*

| **Reference** | **Design & Analyses** | **Association between child externalizing behaviors and parenting practices** | **Significance** | **Other outcomes (e.g., other parent and child variables - control, mediator or moderator variables, and parent mental health)** |
| --- | --- | --- | --- | --- |
| Aydin, 2022 | Cross-sectional. Correlations & Path analysis | *r* = -.26**  Mindful parenting and child externalizing behaviors. | **p* < .05  ***p* < .01 | Mediating role of mindful parenting on the relationship between emotion regulation difficulties in parents and child externalizing behaviors  Parent mental health –Difficulties in Emotion Regulation Scale (DERS) |
| Beer et al., 2013 | Cross-sectional. Pearson’s correlations and Thematic analysis | *r* = -.42*  Mindful parenting and child externalizing behaviors. | **p* < .05  ***p* < .01  ****p* < .001 | Qualitative analysis of mindful parenting practices.  Parent mental health - Parent and  Family Problems subscale of the Questionnaire on  Resources and Stress-Friedrich Short Form (QRS-F) and The Hospital Anxiety and Depression Scale (HADS). |
| Cheung et al., 2019 | Cross-sectional. Correlations & Path analyses | *r* = -.38***  Mindful parenting and child externalizing behaviors. | **p* < .05  ***p* < .01  ****p* < .001 | Moderating and mediating effects of parenting stress son the associations between mindful parenting, parent mental well-being, parents’ affiliate stigma on child externalizing behaviors were also assessed.  Parent mental health –  Parenting Stress Scale (PSS)  and Mental Health Continuum-Short Form (MHC-SF)  *Parents’ characteristics (i.e., disposition to mindful parenting, affiliate stigma, and mental well-being) and parenting stress did not inter-*  *actively explain the variability of children’s behavioral difficulties and prosocial behaviors*. |
|  |  |  |  |  |
| Mills et al., 2022 | Cross-sectional. Spearman-rho correlations and Linear regressions. | *r* = -.31* | ***p* < .01  **p* < .05 | Autism characteristics and child executive function difficulties.  Parent mental health – Stress scale of DASS-21 |
| Raulston et al., 2021 | Cross-sectional. Pearson correlations. | Parenting practices (overall mindful parenting) and externalizing behaviors *r* = -.32**  Parenting practices (low mindful parenting) and externalizing behaviors *r* = -.16 non-significant  Parenting practices (high mindful parenting) and externalizing behaviors *r* = -.29 non-significant | ***p* < .01  **p* < .05 | Autism characteristics.  Parent mental health –  PSI-SF and the Center for Epidemiologic Studies Depression Scale (CES-D) |

**Table 8**

*Mediating or Moderating Effects of Parent Variables.*

| **Mediating and Moderating Effects of Parenting Stress** | | |
| --- | --- | --- |
| **Reference** | **Mediation/Moderation Effects** | **Significance** |
| Cheung et al., 2019 | Moderating and mediating effects of parenting stress on the associations between mindful parenting and child externalizing behaviors. | *β* = − .11 and *p* < .05  Mediator – Parenting Stress  Indirect effect between parents’ disposition to mindful parenting and children’s externalizing behaviors through the mediating effects of parenting stress.  There were no significant moderating effects. |
| Osborne et al., 2008 | Mediating effects of parenting practices and parenting stress on child externalizing behaviors. | *β* = − .414*** and *p* < .001  Mediator – Parenting Stress  The association between the parenting practices - limit setting at baseline and child externalizing behaviors at follow-up continued to be a strong significant association, through the mediating effects of parenting stress. |
| **Mediating and Moderating Effects of Other Parent Variables** | | |
| **Reference** | **Mediation/Moderation Effects** | **Significance** |
| Aydin, 2022 | Mediating role of mindful parenting | (β = −.198, β = 0.386, β = -.238; p < 0.05, respectively.  Mediator - Mindful parenting  The effects between emotion regulation difficulties and externalizing behaviors were partially mediated by mindful parenting. Emotion regulation difficulties significantly predicted mindful parenting and externalizing behaviors, while mindful parenting significantly predicted externalizing behaviors |
| Boonen et al., 2014 | Moderator effects of parenting practices. | No moderating effects were found. |
| Cheung et al., 2019 | Moderating and mediating effects of parent’s mental well-being. | *β* = -.13 and *p* > .05  Mediator – Parent mental well-being.  Indirect effect between parenting stress and children’s externalizing behaviors, through the mediating effects of parent’s mental well-being.  No moderating effects were found. |
| Greenlee et al., 2022~~0~~ | Mediating effects of parenting practices. | Mediator – Parenting practices.  Parenting style - Authoritarian parenting mediated the effect of relationship satisfaction on child externalizing behaviors. |
| Lindsey et al., 2020 | Moderator effects of parenting practices | Moderator – Parenting practices.  Lower externalizing behaviors for children with higher levels of autistic characteristics was associated with higher levels of parenting practice – negative.  Higher externalizing behaviors for children with lower levels of autistic characteristics was associated with higher levels of parenting practice – negative. |
| Osborne et al., 2008 | Mediating effects of parenting practices. | Mediator – Parenting practices.  The relationship between parenting stress at baseline and child externalizing problems was no longer existent at follow-up, when parenting practice of limit setting mediated the relationship. |
| Shawler & Sullivan, 2017 | Mediating effects of parenting practices | Mediator – Parenting practices.  Parenting practice - discipline mediated the associations between parenting stress and frequency of child externalizing behaviors.  Parenting practice - discipline mediated the associations between parent stress and child externalizing behaviors.  Parenting practice - harsh and over corrective mediated the relationship between parent stress and child externalizing behaviors. |
